# A Q-Learning-Enhanced Cuckoo Catfish Optimizer (CCO-RL): A Comparative Study of Nine Metaheuristics Applied to CEC2017, CEC2022 and Engineering Design Problems

**DOI:** 10.3390/biomimetics11060422

**Published:** 2026-06-14

**Authors:** Arar Al Tawil, Amnah Alshahrani, Bilal Ibrahim Alqudah, Hana Fathi

**Affiliations:** 1 Department of Computer Science, Faculty of Information Technology, Applied Science Private University, Amman 11937, Jordan; 2 Department of Information Systems, College of Computer and Information Sciences, Princess Nourah bint Abdulrahman University, P.O. Box 84428, Riyadh 11671, Saudi Arabia; aaalshahrani@pnu.edu.sa; 3 Cybersecurity and Cloud Computing Department, Ajloun National University, Ajloun 26810, Jordan; balqudah@anu.edu.jo; 4 College of Information Technology, Amman Arab University, Amman 11953, Jordan; h.abuhashim@aau.edu.jo

**Keywords:** Cuckoo Catfish Optimizer, Q-learning, reinforcement learning, adaptive operator selection, metaheuristic optimization, CEC2017, CEC2022

## Abstract

The Cuckoo Catfish Optimizer (CCO) is a recent swarm method with four built-in movement strategies. Its weakness is not the moves themselves but the way it chooses among them: a fixed chain of random-versus-threshold (rand>C) tests that ignores how each agent is actually doing and keeps no memory of which move has been paying off. On harder, higher-dimensional problems, this rigidity drains diversity and the search stalls. We propose CCO-RL, which hands the choice of move to a small tabular Q-learning controller. For every agent at every iteration, the controller reads a 48-state summary of the agent’s crowding, its recent stagnation and how far the run has progressed, then picks one of the four moves. A bounded reward and a decaying ε-greedy rule let it learn a policy online with no extra function evaluations. We test CCO-RL against the original CCO and eight popular metaheuristics on CEC2017 (D=30,50) and CEC2022 (D=20): 70 instances, 30 runs each. CCO-RL earns the best overall Friedman rank (1.69) and significantly beats every external competitor according to the Nemenyi test. It also finds the best mean design in three engineering problems.

## 1. Introduction

Optimization sits at the heart of a huge range of problems in engineering and science, including controller tuning, model training, mechanical part sizing and power schedule planning [1,2]. Gradient- and Newton-based methods deal with smooth, convex problems very well, but real problems are rarely so well behaved; they tend to be bumpy and non-differentiable, high-dimensional, costly to evaluate and littered with local optima. Population-based metaheuristics are a pragmatic answer to this; they ask nothing about gradients or smoothness, and, given a reasonable evaluation budget, they can get close to the global optimum of almost any problem [1,3].

The past three decades have produced a rich family of nature-inspired metaheuristics, from Genetic Algorithms (GA) [4], Particle Swarm Optimization (PSO) [5] and Differential Evolution (DE) [6] to the Grey Wolf Optimizer (GWO) [7], the Whale Optimization Algorithm (WOA) [8], Harris Hawks Optimization (HHO) [9], the Equilibrium Optimizer (EO) [10], the RUN optimizer [11], the recent Fungal Growth Optimizer (FGO) [12] and the cuckoo search lineage of Lévy flight optimizers [13]. Most recently, Wang et al. proposed the *Cuckoo Catfish Optimizer* (CCO) [14], a swarm-based optimizer inspired by the brood-parasitic behavior of cuckoos and the foraging dynamics of catfish. The CCO is unusually rich in movement operators, and each agent can be updated by the following: a chaotic predation rule that drives it toward the best individual with Cauchy-modulated steps; logarithmic spiral and spherical surround search rules; a compressed-space exploration rule; and a death-and-parasitism mechanism that re-injects diversity. The original paper reports competitive results on classical benchmark functions and engineering problems. We single out the CCO as the basis for this study for three reasons: First, it is very recent and has not yet been widely improved, which leaves clear room for a contribution. Second, and more importantly, it is unusually rich in built-in search behaviors; where most metaheuristics expose only one or two operators, the CCO already contains four genuinely different moves, which makes it an ideal platform for studying *how* to choose among operators rather than *which* new operator to design. Third, as a bio-inspired optimizer whose weakness lies purely in its selection logic rather than in the moves themselves, it is a clean test case for an adaptive, learning-based controller.

When we ran the original CCO on CEC2017 at D=30 and higher, though, a clear pattern emerged: the swarm loses diversity late in the run and gets stuck on the hybrid and composition functions. The culprit is the way the CCO decides which move to make: that decision comes from a nested chain of rand>C tests, with C=1−t/T shrinking steadily as the run goes on. Therefore, which branch an agent takes (predation, one of the two surround searches, the transition step or compressed-space exploration) is essentially set by the clock and a few coin flips. The agent’s own situation never enters into it, and it does not matter whether the agent is leading the pack or stuck in a rut, sitting in a crowd or out on its own. Nor is there any memory. A move that helped early on keeps being chosen with the same schedule-driven odds long after it has stopped helping. This is the familiar trap of single-schedule metaheuristics, where one fixed balance of exploration and exploitation rarely serves the whole run [2,15].

A growing body of work shows that *adaptive operator selection* (AOS) can substantially mitigate this problem by dynamically choosing, at each iteration and possibly for each agent, among a pool of candidate operators on the basis of their observed historical performance [16]. Reinforcement learning (RL) is a particularly natural fit for AOS because operator selection can be framed as a sequential decision problem: states encode the current condition of the agent, actions correspond to candidate operators and rewards correspond to the fitness improvement obtained after applying the chosen operator [17]. Tabular Q-learning [18,19] is attractive in this setting because it is model-free and off-policy and has well-understood convergence properties. Recent studies have integrated Q-learning or related RL methods into PSO [20], DE [21,22], GWO-based control [23] and other metaheuristic families [24,25], consistently reporting improved robustness.

Motivated by these observations, this paper proposes CCO-RL, a Q-learning-enhanced variant of the CCO in which the fixed rand>C strategy cascade is replaced by a single shared tabular Q-learning controller. The controller observes, for each individual, a discrete state that encodes its aggregation degree relative to the swarm, its per-individual stagnation level and the current phase of the search, and it selects one of four core movement strategies. The reward is a bounded, scale-invariant function of the relative fitness improvement, and the policy is ε-greedy with linear exploration decay. Boundary handling, death-and-parasitism, Lévy steps and all other constants are inherited unchanged from the CCO, so the only altered mechanism is strategy selection and the per-iteration function evaluation budget is preserved.

The main contributions of this paper are as follows:

1. We propose CCO-RL, which integrates a shared tabular Q-learning controller into the CCO for state-conditioned, memory-based strategy selection, without increasing the per-iteration function evaluation budget.
2. We compare CCO-RL against the original CCO and eight state-of-the-art metaheuristics (GA, PSO, DE, GWO, HHO, RUN, EO, FGO) applied to CEC2017 at D=30 and D=50, CEC2022 at D=20 (70 instances, 30 runs each) and three constrained engineering design problems. CCO-RL attains the lowest overall Friedman mean rank (1.69) and is significantly better than all eight external competitors according to the Nemenyi post hoc test.
3. We perform a parameter sensitivity analysis of the three Q-learning hyperparameters (α, γ, $\varepsilon_{\mathrm{end}}$) and an ablation study considering the components of the state encoding and the controller, showing that CCO-RL is robust to its settings and that adaptive state-based selection is the principal driver of the gains.

The remainder of this paper is organized as follows: Section 2 surveys related work on metaheuristics and reinforcement learning-based operator selection. Section 3 reviews the original CCO. Section 4 describes CCO-RL in full detail. Section 5 presents the experimental setup, parameter sensitivity analysis and ablation study. Section 6 reports the comparative results with statistical analysis. Section 7 evaluates the engineering design problems. Section 8 discusses the findings, and Section 9 concludes.

## 2. Related Work

Population-based metaheuristics have grown into one of the busiest corners of computational intelligence, and it is helpful to set CCO-RL against this backdrop before describing it. The field rests on a handful of classics: Genetic Algorithms [4] from biology for borrowed selection, crossover and mutation; Particle Swarm Optimization [5] for modeling the social behavior of bird flocks; Differential Evolution [6], which introduced its now-famous difference vector mutation; and Ant Colony Optimization [26], which shows how stigmergy could attack combinatorial problems. These methods are still strong baselines, and DE in particular remains hard to beat in constrained problems. What followed was a long wave of newer, often metaphor-driven optimizers, including the Grey Wolf Optimizer [7], the Whale Optimization Algorithm [8], Harris Hawks Optimization [9], the Equilibrium Optimizer [10], the RUN optimizer [11], and, very recently, the Fungal Growth Optimizer [12]. The Cuckoo Catfish Optimizer [14] belongs to this newer generation and to the cuckoo search lineage that began with cuckoo search [13]. Across this whole family, two criticisms keep coming back: many algorithms differ mostly in their metaphor rather than in their actual search behavior, and most rely on hand-tuned schedules that fix the balance between exploration and exploitation in advance [2,15].

Because no single algorithm wins everywhere, a large body of work has tried to improve existing optimizers rather than invent new ones. The usual recipes include hybridizing two algorithms so their strengths complement each other, seeding the starting population with opposition-based or chaotic sampling to widen early coverage and swapping fixed control parameters for adaptive or self-adaptive rules. The CCO itself belongs to this tradition, and it stitches several distinct moves—a chaotic predation rule, two surround searches, a compressed-space step and a death-and-parasitism mechanism—into a single optimizer. The catch is that bundling good operators together does not, on its own, tell the algorithm when to use each one; that decision is usually handed to a fixed schedule, and that is precisely the gap our work targets.

A more principled answer to the question of when to use each operator is provided by adaptive operator selection (AOS). The idea is to keep a pool of candidate operators and choose among them on the fly, favoring the ones that have recently produced improvements. Early AOS schemes framed the choice as a multi-armed bandit and reported clear gains over static selection [16]. The appeal is that the algorithm no longer has to commit to one exploration–exploitation trade-off for the whole run; it can shift its preferences as the search moves from a broad early phase into a fine late one. AOS does raise its own questions, though: Which signal should drive the reward? How much memory should the controller keep? Should the choice be made once for the whole population or separately for each individual?

Reinforcement learning provides a natural language for exactly those questions, and pairing it with metaheuristics has become an active topic in its own right [24]. Q-learning [17,18] is the most common tool because it is model-free, cheap and easy to bolt onto an existing optimizer. Researchers have used it to tune parameters and pick operators inside Particle Swarm Optimization [20], Differential Evolution [21,22], Grey Wolf-Based controllers [23] and Sand Cat Swarm Optimization [25], and they have consistently reported steadier, more robust behavior. The designs differ mostly in three places: the state (often the iteration phase, a diversity measure or a fitness band), the action set (a parameter value or an operator) and the reward (usually tied to the fitness improvement). A recurring limitation is that many of these methods steer a single parameter, or pick from a small two-operator menu, and they apply the same decision to the entire swarm at once rather than to each agent.

CCO-RL is positioned squarely in this space, but it makes a few deliberate choices that set it apart from earlier RL-enhanced optimizers. Instead of tuning a parameter, it selects among the four movement strategies that the CCO already provides, so the learning sits on top of a genuinely diverse operator pool. The decision is taken per agent and per iteration, conditioned on a compact picture of that agent’s situation (how crowded it is, how long it has gone without improving and how far the run has progressed). The whole controller is a small shared lookup table updated by ordinary tabular Q-learning, so it adds no function evaluations and a negligible amount of memory. In short, where much prior work has used reinforcement learning to nudge a parameter, we use it to govern strategy selection inside a metaheuristic that is already rich in strategies but poor at choosing among them. The experiments that follow test whether that one change is enough to lift the CCO from being a competitive method to being a leading one, and we report a comparison against nine other algorithms using the nonparametric statistical protocol recommended for this kind of study [27].

## 3. The Cuckoo Catfish Optimizer (CCO)

The CCO [14] is a swarm-based metaheuristic in which each candidate solution $x_{i}\in R^{D}$ represents the position of an individual in a search space bounded by lb and ub. The population is initialized uniformly at random, and each individual is assigned logarithmic spiral coordinates used later by the surround search operators. At each iteration *t*, the algorithm maintains two global schedule variables:

(1) $$C=1-\frac{t}{T},\;T_{f}={\left(1-\sin\frac{\pi t}{2T}\right)}^{t/T},$$

where *C* decays linearly from 1 to 0 and $T_{f}$ is a nonlinear amplitude factor. Two aggregation measures quantify how concentrated the swarm is: the population-level degree Dis and the per-individual degree $J_{i}$,

(2) $$\mathrm{Dis}=|\bar{\frac{x_{i}-x^{*}}{x^{w}-x^{*}+\epsilon }}|,\;J_{i}=|\;\mathrm{mean}\;\frac{x_{i}-x^{*}}{x^{w}-x^{*}+\epsilon }|,$$

where $x^{*}$ and $x^{w}$ are the best and worst individuals and ϵ avoids division by zero.

### 3.1. Movement Strategies

For each agent, the CCO draws a uniform scalar and compares it against *C* in a nested cascade that selects one of the following moves: the *chaotic predation* move, which drives the agent toward the best individual with a Cauchy-modulated, time-decaying step whose exact form depends on $J_{i}$ relative to Dis; the *spiral surround search*, which spirals the agent around a learning anchor using its pre-computed logarithmic spiral coordinates; the *spherical surround search*, which rotates the agent around one of the three best individuals or the swarm mean using random spherical angles and an adaptive radius; or the *compressed-space exploration* move, which recombines the agent from random peers and the best individual for diversity. After the move, a death-and-parasitism step re-initializes the agent with small probability (either uniformly in the domain or near the best position through a Lévy-modulated egg-laying rule [28]), the position is repaired to the bounds and a greedy selection step accepts the new position only if it improves fitness.

### 3.2. Structural Observations

Three properties of the CCO motivate this work: First, the choice among the four strategies is made by a nested cascade of rand>C tests. The branch taken depends only on the global schedule *C* and chance, not on whether the individual is elite or stagnating. Second, there is no operator memory, meaning no per-agent state, no credit assignment and no mechanism to learn which strategy works best for which type of agent. Third, the CCO tracks stagnation with global counters shared by the whole swarm, so an individual that is personally stuck cannot trigger a tailored response. CCO-RL addresses all three points by replacing the cascade with a state-conditioned, memory-based controller.

## 4. The Q-Learning-Enhanced CCO (CCO-RL)

CCO-RL preserves the population dynamics, the four movement strategies, the death-and-parasitism mechanism and the function evaluation budget of the CCO (*N* evaluations per iteration) but replaces the fixed rand>C cascade with a single shared tabular Q-learning controller that selects, for every individual at every iteration, one of four candidate strategies on the basis of a discrete search state.

### 4.1. State Encoding

Each individual *i* is mapped to 1 of 48 discrete states. The first feature is the individual aggregation degree $J_{i}$ of Equation (2) relative to Dis, discretized into four buckets:


(3) $$s_{J}=\left\{\begin{array}{cc} 0, & J_{i}<0.5\;\mathrm{Dis},\\ 1, & 0.5\;\mathrm{Dis}\leq J_{i}<\mathrm{Dis},\\ 2, & \mathrm{Dis}\leq J_{i}<2\;\mathrm{Dis},\\ 3, & J_{i}\geq 2\;\mathrm{Dis}. \end{array}\right.$$

The second is a per-individual no-improvement (stagnation) counter ${\mathrm{stag}}_{i}$, discretized as $s_{\mathrm{stag}}=0,1,2,3$ for ${\mathrm{stag}}_{i}=0$, <5, <15 and ≥15, respectively. The third is the search phase, $s_{\mathrm{iter}}=0,1,2$, for the early, middle and late thirds of the run. The composite state is

(4) $$s_{i}=s_{J}\cdot 12+s_{\mathrm{stag}}\cdot 3+s_{\mathrm{iter}},$$

giving 4×4×3=48 states. Replacing the CCO’s global counters by per-individual stagnation tracking is what makes a tailored, agent-specific response possible. All three features are computed *deterministically* from the current population at every iteration: the aggregation degree $J_{i}$ from the agent’s position relative to the best and worst individuals (Equation (2)), the stagnation counter from the agent’s own accept/reject history and the search phase from the iteration index. They are, therefore, neither random nor driven by a fixed cyclic schedule; the only stochastic element in the controller is the ε-greedy exploration of Equation (5), whose randomness decays over the run.

### 4.2. Action Set

The controller chooses among four actions, each a self-contained movement strategy inherited from the CCO: a=0 chaotic predation; a=1 spiral surround search; a=2 spherical surround search; a=3 compressed-space exploration. The same four strategies are available to every individual at every iteration; it is the controller, not the *C* schedule, that decides which one to apply.

### 4.3. Q-Learning Machinery

CCO-RL maintains a single shared Q-table $Q\in R^{48\times 4}$ initialized to 0. Action selection follows an ε-greedy policy with linearly decaying exploration:


(5) $$a_{i}=\left\{\begin{array}{cc} \mathrm{uniform}\;\mathrm{random}, & \mathrm{with}\;\mathrm{prob}.\;\varepsilon (t),\\ \arg\max_{a^{\prime }}Q\left[s_{i},a^{\prime }\right], & \mathrm{otherwise}, \end{array}\right.\;\varepsilon \left(t\right)=\varepsilon_{\mathrm{start}}+\left(\varepsilon_{\mathrm{end}}-\varepsilon_{\mathrm{start}}\right)\frac{t}{T-1}.$$

**Figure 1:**
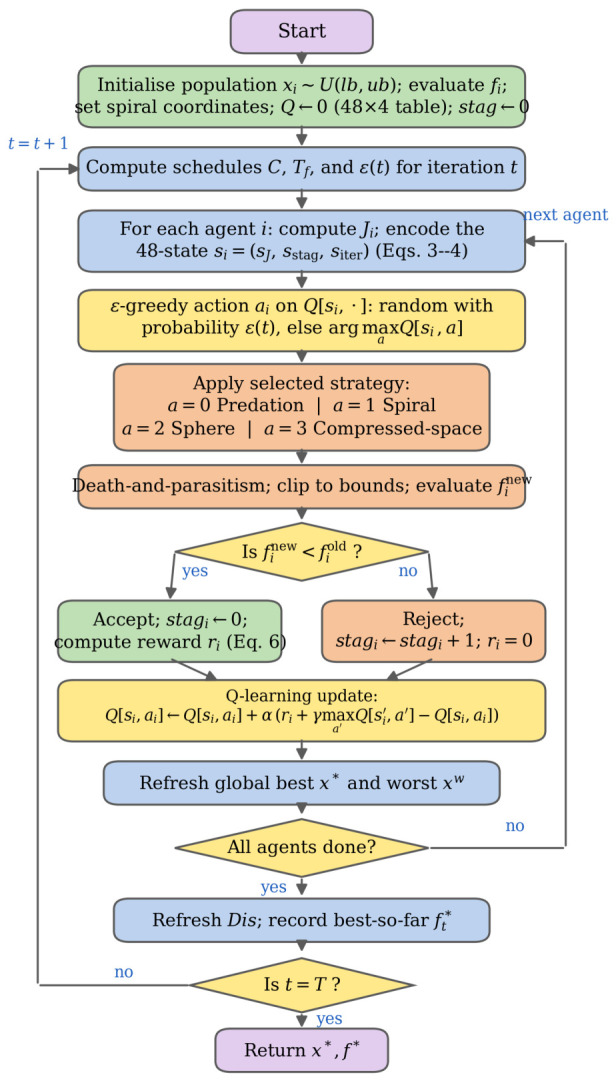
Flowchart of the proposed CCO-RL. The inner loop (right-hand branch) iterates over the *N* agents; the outer loop repeats for *T* iterations. The shaded action block is the only point where the Q-learning controller intervenes, replacing the CCO’s fixed rand>C cascade.

After the chosen strategy is applied, the position is repaired and evaluated, and a bounded, scale-invariant reward is computed from the relative improvement:

(6) $$r_{i}=\mathrm{clip}\;\left(\frac{f_{i}^{\mathrm{old}}-f_{i}^{\mathrm{new}}}{|f_{i}^{\mathrm{old}}|+10^{-12}},\;0,\;1\right),$$

which is 0 when the move fails to improve the individual and a bounded positive value otherwise. The Bellman update is

(7) $$Q\left[s_{i},a_{i}\right]\leftarrow Q\left[s_{i},a_{i}\right]+\alpha \left(r_{i}+\gamma \max_{a^{\prime }}Q\left[s_{i}^{\prime },a^{\prime }\right]-Q\left[s_{i},a_{i}\right]\right),$$

with learning rate α=0.1, discount factor γ=0.9 and exploration schedule $\left(\varepsilon_{\mathrm{start}},\varepsilon_{\mathrm{end}}\right)=\left(1.0,0.1\right)$. The complete procedure is given in Algorithm 1 and illustrated as a flowchart in Figure 1.

The three hyperparameters play distinct, well-understood roles in Equations (5)–(7): The learning rate α∈(0,1] weights how strongly each new observation revises the stored value $Q[s_{i},a_{i}]$, where a small α gives slow but stable averaging, which suits the noisy, scale-varying rewards of Equation (6), where one lucky improvement should not dominate the estimate. The discount factor γ∈[0,1) sets how much the controller values the best achievable value of the next state, $\max_{a^{\prime }}Q\left[s_{i}^{\prime },a^{\prime }\right]$, relative to the immediate reward, where a high γ pushes the policy to favor moves that keep an agent in promising regions rather than chasing one-step gains. The exploration rate ε(t) governs the exploration–exploitation balance of action selection. It starts at 1.0, so every strategy is tried in every state, and decays linearly to a small residual $\varepsilon_{\mathrm{end}}$, after which the controller mostly commits to the actions it has learned to trust. The empirical preference for a small learning rate and low residual exploration, confirmed in Section 5.3, is designed to exactly reflect the following: learning cautiously from noisy feedback, then exploiting the converged policy late in the run. Finally, following standard practice for a fair comparison, the hyperparameters of every competitor in Table 1 are taken from its original publication. For CCO-RL, we use the values above, which the sensitivity analysis shows to be near-optimal and robust to perturbation; therefore, the comparison does not hinge on a particular tuning under the present experimental conditions.
**Algorithm** **1** Q-Learning-Enhanced Cuckoo Catfish Optimizer (CCO-RL)**Require: ***N*, *T*, lb, ub, *f*, α=0.1, γ=0.9, $\varepsilon_{\mathrm{start}}=1.0$, $\varepsilon_{\mathrm{end}}=0.1$ 1:Initialise $x_{i}∼U\left(\mathrm{lb},\mathrm{ub}\right)$; evaluate $f_{i}$; set spiral coordinates 2:$Q\leftarrow 0_{48\times 4}$; $\mathrm{stag}\leftarrow 0_{N}$; compute $x^{*},x^{w},\mathrm{Dis}$ 3:**for** t=1 to *T* **do** 4:    Compute *C*, $T_{f}$ and ε(t) 5:    **for** i=1 to *N* **do** 6:        Compute $J_{i}$; encode state $s_{i}$ (Equations (3) and (4)) 7:        $a_{i}\leftarrow \varepsilon$-greedy on $Q[s_{i},\cdot ]$ 8:        Apply strategy $a_{i}$; death-and-parasitism; clip; evaluate $f_{i}^{\mathrm{new}}$ 9:        **if** $f_{i}^{\mathrm{new}}<f_{i}^{\mathrm{old}}$ accept, ${\mathrm{stag}}_{i}\leftarrow 0$; **else** ${\mathrm{stag}}_{i}\leftarrow {\mathrm{stag}}_{i}+1$10:        Compute reward (Equation (6)); encode $s_{i}^{\prime }$; update *Q* (Equation (7))11:        Refresh $x^{*},x^{w}$12:    **end for**13:    Refresh Dis; record $f_{t}^{*}$14:**end for**15:**return** $x^{*}$, $f^{*}$, convergence curve

### 4.4. Computational Complexity

Each iteration of the CCO performs one population sort (O(NlogN)), *N* objective evaluations ($O(Nc_{f})$) and O(ND) vector updates, so its total cost over *T* iterations is $O(T\left(N\log N+Nc_{f}+ND\right))$, dominated by the objective evaluations. CCO-RL adds only one state computation per individual (O(D) for $J_{i}$), one Q-table lookup and one Q-table update per individual (O(1) since |A|=4) and the storage of a single 48×4 table. The per-iteration overhead is therefore O(ND), asymptotically dominated by the evaluation cost, so CCO-RL has the same Big-O complexity as the CCO. The additional memory, 192 floats, is negligible.

## 5. Experimental Setup

### 5.1. Benchmark Suites

We use two competition suites: CEC2017 [29], which comprises 29 functions (unimodal F1, F3, simple multimodal F4–F10, hybrid F11–F20, composition F21–F30, with the unstable F2 being excluded as recommended) tested at D=30 and D=50; CEC2022 [30], which comprises 12 functions at D=20. Together, these give 70 benchmark instances. The search domain is ${\left[-100,100\right]}^{D}$. We additionally study how CCO-RL scales to D=100 on CEC2017 against the base CCO (Section 6.7) and evaluate three constrained engineering design problems (Section 7).

### 5.2. Compared Algorithms and Configuration

CCO-RL is compared against the original CCO [14] and eight widely used metaheuristics spanning evolutionary, swarm, physics-based and recent families: GA [4], PSO [5], DE [6], GWO [7], HHO [9], RUN [11], EO [10] and FGO [12]. All algorithms use a population of N=30, a budget of T=500 iterations and R=30 independent runs per instance under paired random seeds. Algorithm-specific hyperparameters are taken from each algorithm’s original paper, as summarized in Table 1. Experiments are implemented in Python 3 (NumPy and SciPy) and executed in a single-threaded manner so that the timings are comparable across algorithms. All runs are performed on the same workstation equipped with an Intel Core i7-12700H processor (2.3 GHz) and 16 GB of RAM running Windows and Python 3.11. Because every algorithm shares this environment, the runtimes reported in Section 6.8 can be compared directly.

### 5.3. Parameter Sensitivity Analysis

**Figure 2:**
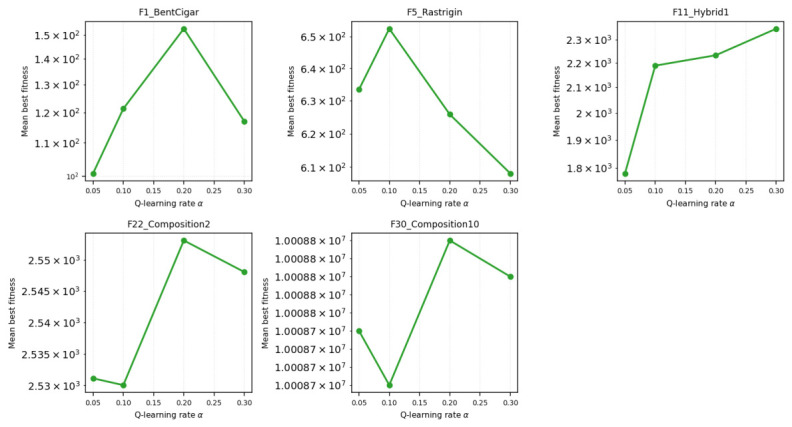
Parameter sensitivity of CCO-RL to the Q-learning rate α (mean best fitness over 30 runs, log scale).

**Figure 3:**
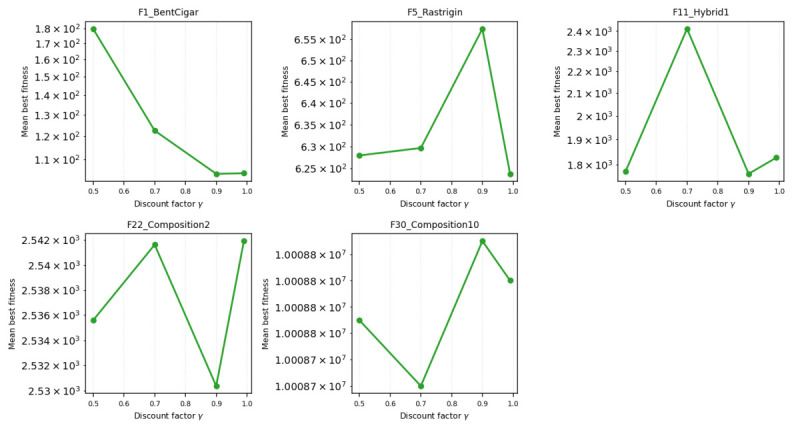
Parameter sensitivity of CCO-RL to the discount factor γ.

**Figure 4:**
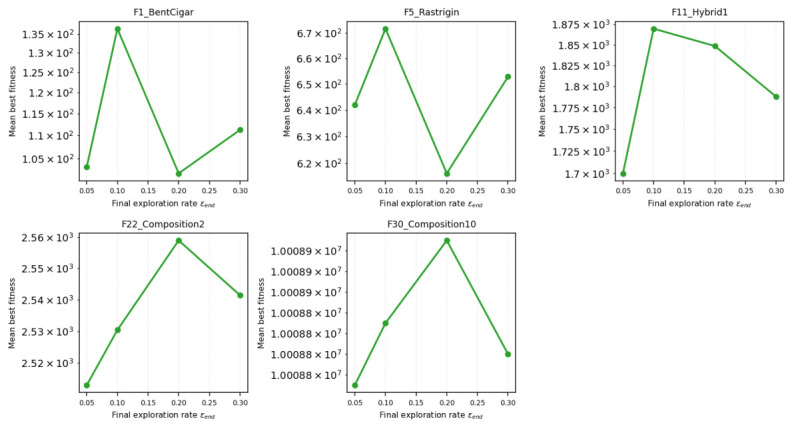
Parameter sensitivity of CCO-RL to the final exploration rate $\varepsilon_{\mathrm{end}}$.

The CCO-RL controller introduces three hyperparameters: the Q-learning rate α, the discount factor γ and the final exploration rate $\varepsilon_{\mathrm{end}}$. We sweep each independently on five representative CEC2017 functions at D=30 while holding the others at their defaults. For α∈{0.05,0.10,0.20,0.30}, the Friedman mean ranks are 1.8, 2.2, 3.4 and 2.6 (Figure 2); for γ∈{0.50,0.70,0.90,0.99}, they are 2.4, 2.8, 2.2 and 2.6, a spread of only 0.6 (Figure 3); and, for $\varepsilon_{\mathrm{end}}\in \left\{0.05,0.10,0.20,0.30\right\}$, they are 1.4, 3.4, 2.6 and 2.6 (Figure 4). Small learning rates and low residual exploration are preferred, and the default configuration achieves near-optimal ranks on every sweep.

### 5.4. Strategy Effectiveness (Ablation Study)

**Figure 5:**
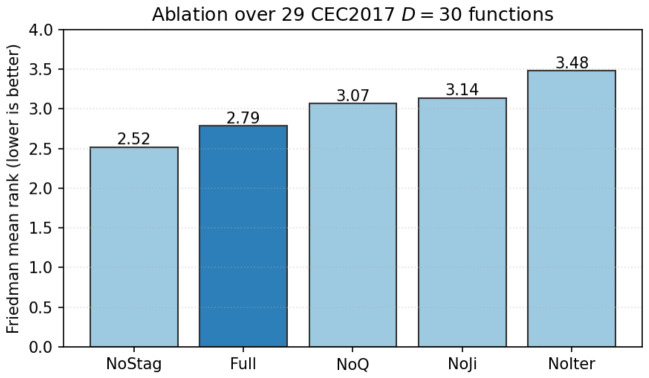
Ablation study: Friedman mean rank of CCO-RL and its four reduced variants on five representative CEC2017 D=30 functions. Lower is better.

To isolate the contribution of each component of the controller, we construct the following four reduced variants and compare them against the full CCO-RL on all 29 CEC2017 (D=30) functions: NoQ (Q-learner replaced by uniform random action selection), NoJi (aggregation feature dropped), NoStag (stagnation feature dropped) and NoIter (search-phase feature dropped). Table 2 reports the mean best fitness and standard deviation of every variant over the 30 runs, and Figure 5 visualizes the mean ranks. The full encoding is competitive across the suite, attaining the best Friedman rank on the unimodal functions and ranking second overall (2.79), behind only NoStag (2.52). The full Q-learning controller also outperforms uniform random action selection (NoQ, 3.07) overall, which indicates that the learned policy, rather than mere strategy diversity, drives the behavior. At the same time, an omnibus Friedman test across the five variants is not significant ($\chi^{2}=6.18$, p=0.19), so no single variant is reliably better or worse than the others. We therefore read this study as evidence that CCO-RL is robust to the precise choice of state features rather than fragile to it: removing any one feature leaves performance statistically unchanged, while the full encoding remains among the best performers and is the only design that requires no prior decision about which features to discard.

## 6. Benchmark Results

This section reports the comparative performance of CCO-RL, the original CCO and the eight competitors. All results are aggregated over 30 paired runs per instance, and the best mean per function is shown in bold.

### 6.1. CEC2017 at D = 30

**Figure 6:**
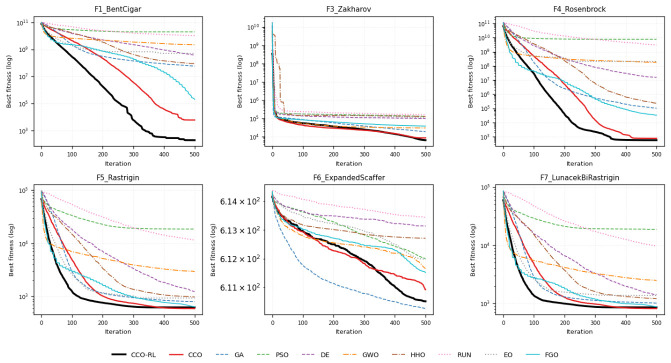
Mean convergence curves on CEC2017 (D=30), functions F1–F7. Each of the ten algorithms is drawn with a distinct color and line style (see legend); CCO-RL is the bold black curve. Mean over 30 runs, logarithmic scale.

**Figure 7:**
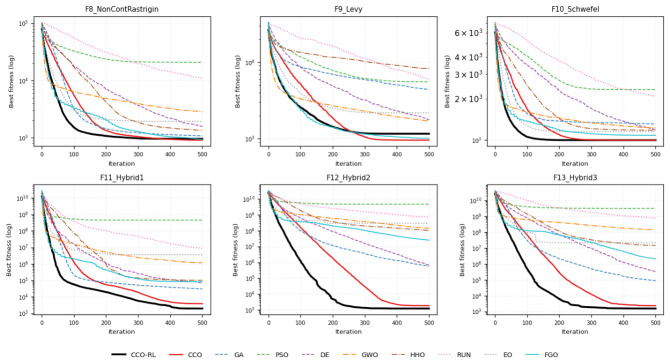
Mean convergence curves on CEC2017 (D=30), functions F8–F13. Each of the ten algorithms is drawn with a distinct color and line style (see legend); CCO-RL is the bold black curve. Mean over 30 runs, logarithmic scale.

**Figure 8:**
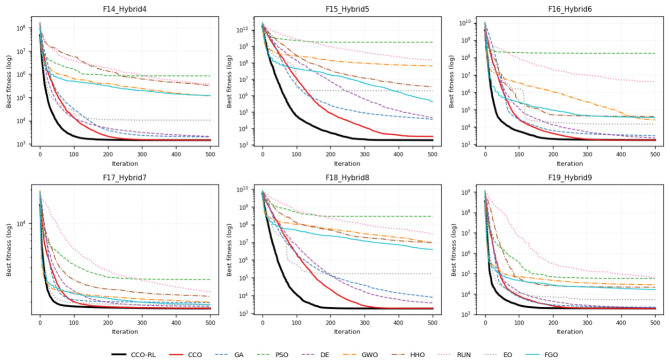
Mean convergence curves on CEC2017 (D=30), functions F14–F19. Each of the ten algorithms is drawn with a distinct color and line style (see legend); CCO-RL is the bold black curve. Mean over 30 runs, logarithmic scale.

**Figure 9:**
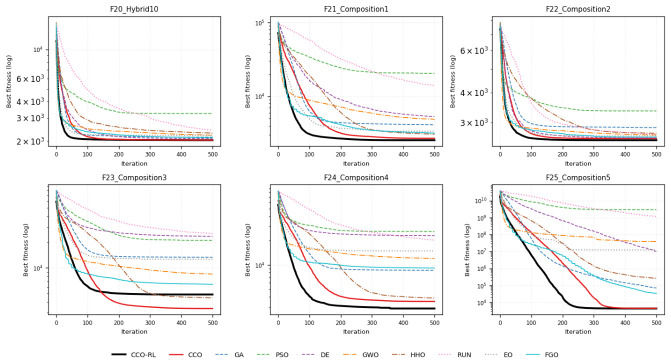
Mean convergence curves on CEC2017 (D=30), functions F20–F25. Each of the ten algorithms is drawn with a distinct color and line style (see legend); CCO-RL is the bold black curve. Mean over 30 runs, logarithmic scale.

**Figure 10:**
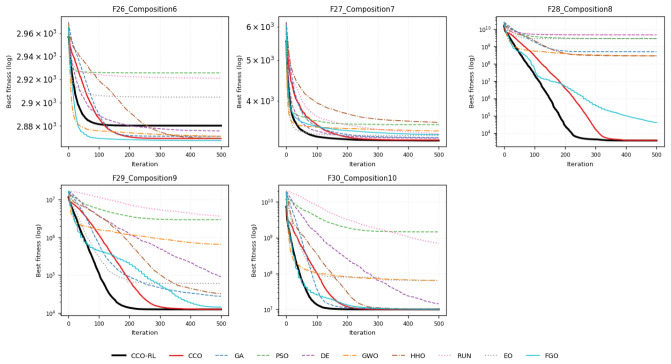
Mean convergence curves on CEC2017 (D=30), functions F26–F30. Each of the ten algorithms is drawn with a distinct color and line style (see legend); CCO-RL is the bold black curve. Mean over 30 runs, logarithmic scale.

Table 3 reports the mean best fitness on the 29 CEC2017 functions at D=30. CCO-RL attains the lowest mean on 19 of 29 functions, and the CCO is best on 8. Its Friedman mean rank is 1.62, the best of all ten algorithms (CCO 1.76, FGO 4.10, GA 4.45). The omnibus Friedman test rejects equal performance ($\chi^{2}=200.3$, $p<10^{-37}$). Convergence curves are shown in Figure 6, Figure 7, Figure 8, Figure 9 and Figure 10.

### 6.2. CEC2017 at D = 50

**Figure 11:**
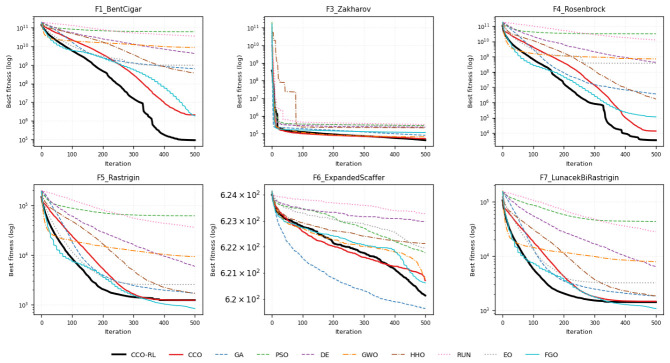
Mean convergence curves on CEC2017 (D=50), functions F1–F7. Each of the ten algorithms is drawn with a distinct color and line style (see legend); CCO-RL is the bold black curve. Mean over 30 runs, logarithmic scale.

**Figure 12:**
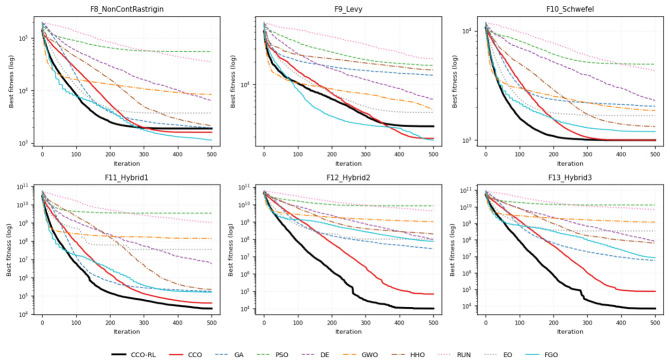
Mean convergence curves on CEC2017 (D=50), functions F8–F13. Each of the ten algorithms is drawn with a distinct color and line style (see legend); CCO-RL is the bold black curve. Mean over 30 runs, logarithmic scale.

**Figure 13:**
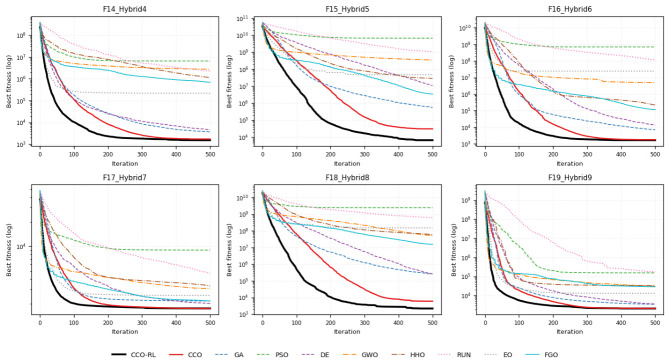
Mean convergence curves on CEC2017 (D=50), functions F14–F19. Each of the ten algorithms is drawn with a distinct color and line style (see legend); CCO-RL is the bold black curve. Mean over 30 runs, logarithmic scale.

**Figure 14:**
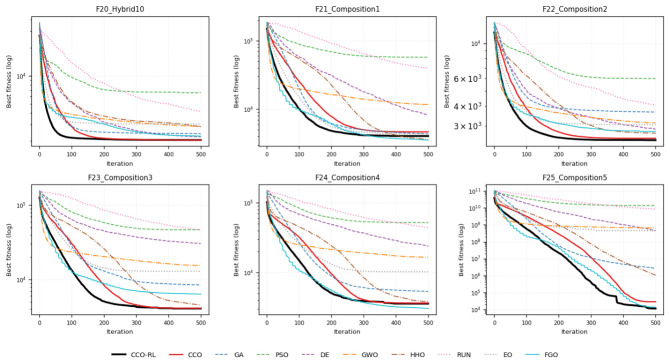
Mean convergence curves on CEC2017 (D=50), functions F20–F25. Each of the ten algorithms is drawn with a distinct color and line style (see legend); CCO-RL is the bold black curve. Mean over 30 runs, logarithmic scale.

**Figure 15:**
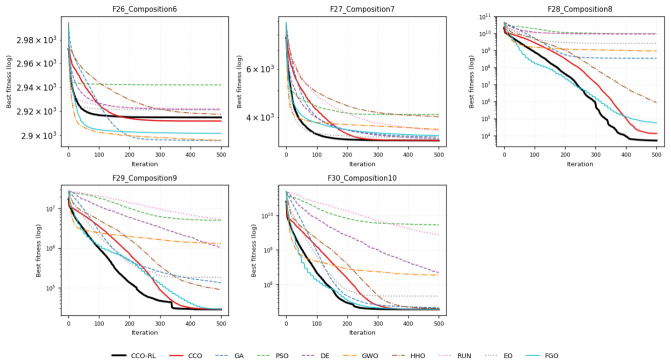
Mean convergence curves on CEC2017 (D=50), functions F26–F30. Each of the ten algorithms is drawn with a distinct color and line style (see legend); CCO-RL is the bold black curve. Mean over 30 runs, logarithmic scale.

At D=50 (Table 4), CCO-RL again attains the lowest mean on 19 of 29 functions and the best Friedman rank (1.59), ahead of the CCO (2.38) and FGO (3.28). The advantage over the classical competitors widens at this dimension: on several hybrid functions, the CCO-RL mean is orders of magnitude lower than that of GA, PSO, GWO and RUN ($\chi^{2}=206.0$, $p<10^{-38}$). Convergence curves are shown in Figure 11, Figure 12, Figure 13, Figure 14 and Figure 15.

### 6.3. CEC2022 at D = 20

**Figure 16:**
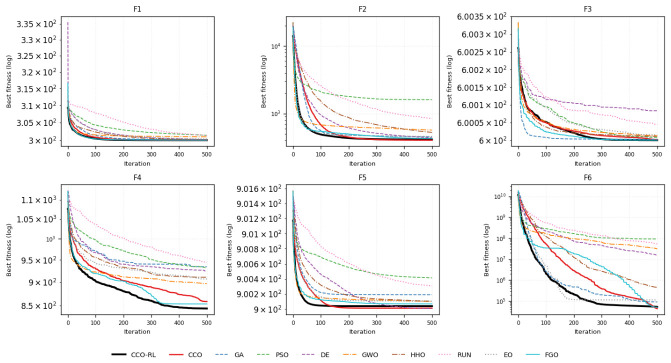
Mean convergence curves on CEC2022 (D=20), functions F1–F6. Each of the ten algorithms is drawn with a distinct color and line style (see legend); CCO-RL is the bold black curve. Mean over 30 runs, logarithmic scale.

**Figure 17:**
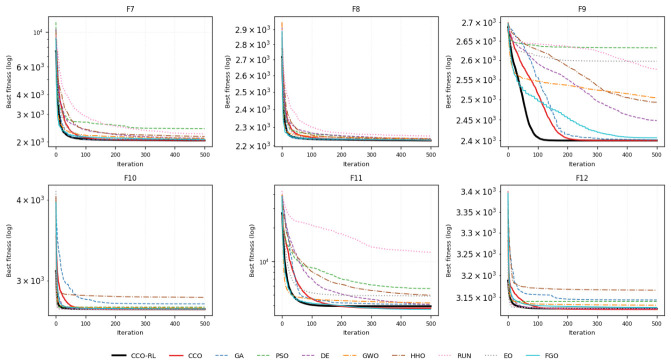
Mean convergence curves on CEC2022 (D=20), functions F7–F12. Each of the ten algorithms is drawn with a distinct color and line style (see legend); CCO-RL is the bold black curve. Mean over 30 runs, logarithmic scale.

Table 5 reports the 12 CEC2022 functions at D=20. Here, CCO-RL and the base CCO are the two strongest algorithms and are very close: the CCO attains the best Friedman rank (1.71) and CCO-RL is a close second (2.12), with both being clearly ahead of the FGO (3.33) and the remaining competitors ($\chi^{2}=69.3$, $p<10^{-10}$). The CEC2022 functions are lower-dimensional and several plateau at the global optimum, which compresses the gap between the two CCO variants. Convergence curves are shown in Figure 16 and Figure 17.

### 6.4. Statistical Analysis

We assess statistical significance with the Friedman test followed by the Nemenyi post hoc test, the standard nonparametric protocol for comparing many optimizers over many problems [27]. The Friedman test is the nonparametric analog of a repeated-measures ANOVA: on each of the *N* benchmark instances, the *k* algorithms are ranked from best (1) to worst (*k*), and each algorithm’s *mean rank* $R_{j}=\frac{1}{N}\sum_{i}r_{i}^{\;j}$ is formed. Under the null hypothesis of equal performance, the statistic $\chi_{F}^{2}=\frac{12N}{k(k+1)}\left(\sum_{j}R_{j}^{2}-\frac{k{\left(k+1\right)}^{2}}{4}\right)$ is approximately $\chi^{2}$-distributed with k−1 degrees of freedom, so a small *p*-value means the algorithms are not equivalent. The mean rank itself is the headline quality score (lower is better). When the omnibus test is significant, the Nemenyi test then declares two algorithms different if their mean ranks differ by more than the critical difference $\mathrm{CD}=q_{\alpha }\sqrt{k(k+1)/(6N)}$.

**Figure 18:**
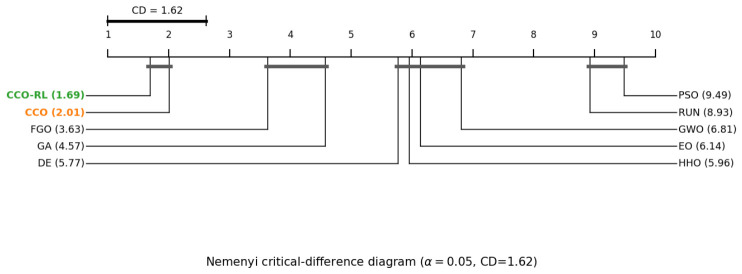
Nemenyi critical difference diagram (α=0.05, CD=1.62). Algorithms joined by a bold bar are not statistically distinguishable. CCO-RL is significantly better than all eight external competitors.

**Figure 19:**
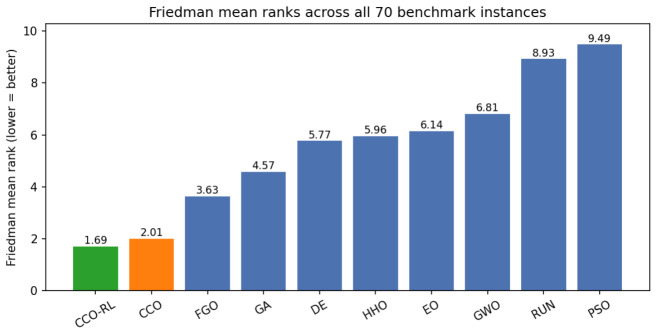
Friedman mean ranks of all ten algorithms across the 70 pooled benchmark instances (lower is better).

Table 6 summarizes the Friedman mean ranks of all ten algorithms on each suite and over the pooled 70 instances. CCO-RL achieves the lowest overall rank (1.69), followed by the CCO (2.01), FGO (3.63), GA (4.57), DE (5.77), HHO (5.96), EO (6.14), GWO (6.81), RUN (8.93) and PSO (9.49). The omnibus test over the 70 instances decisively rejects the null hypothesis of equal performance ($\chi^{2}=466.8$, $p<10^{-90}$). We apply the Nemenyi post hoc test at α=0.05. With k=10 algorithms and N=70 instances, the critical difference is CD=1.62. As Figure 18 shows, CCO-RL is significantly better than all eight external competitors, and is statistically indistinguishable only from the base CCO (1.69 versus 2.01, gap 0.32<CD), which is no surprise given that the two share the same operators. Figure 19 shows the overall ranks graphically.

Per-function Wilcoxon comparisons (Table 7) confirm the same ordering. Against every external competitor, CCO-RL wins on the large majority of the 70 instances (for example, 64/0/6 against PSO, 61/0/9 against RUN and 55/1/14 against the FGO), and the paired Wilcoxon signed-rank test over the pooled means is significant against all eight ($p<10^{-4}$ in every case). The only algorithm CCO-RL does not dominate is its own base CCO, against which it still records more wins than losses overall.

The convergence curves make the ranking visible at the trajectory level. On the unimodal and hybrid functions (e.g., F1 and F11–F18), CCO-RL (bold black) separates from the field within the first 50–100 iterations and keeps descending, while PSO, RUN and the GWO flatten early. This is the signature escaping from the local optima that traps fixed-schedule methods. On the composition functions, the curves run closer together, consistent with the smaller rank gaps there, yet CCO-RL and the CCO still finish at or below every external competitor. The base CCO tracks CCO-RL most closely, as expected, since the two share operators, but the CCO-RL curve sits consistently lower through the mid-to-late phase, which is exactly where the learned policy tilts toward exploitation.

### 6.5. Learned Strategy Selection Policy

**Figure 20:**
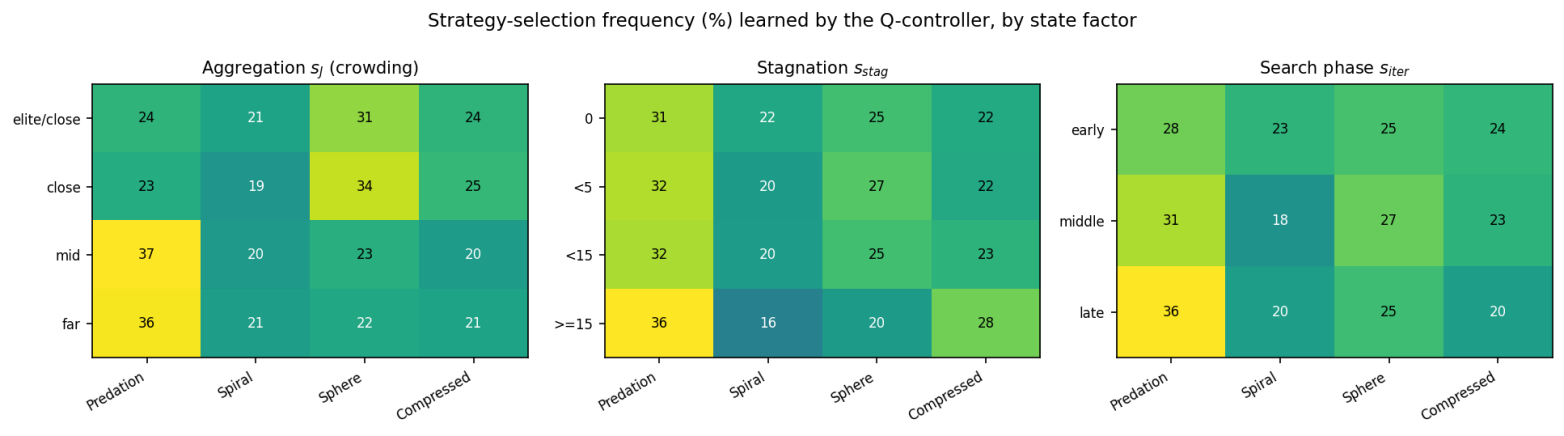
Strategy selection frequency (%) learned by the Q-controller, aggregated by each state factor: crowding $s_{J}$ (**left**), stagnation $s_{\mathrm{stag}}$ (**center**) and search phase $s_{\mathrm{iter}}$ (**right**). Computed over $1.5\times 10^{5}$ logged selections on five representative CEC2017 (D=30) functions.

A natural question pertains to *what* the controller actually learns: Which of the four strategies does it come to prefer, and in which situations? To answer this, we instrument CCO-RL to log every (state, action) selection over 10 runs on the five representative functions of Section 5.4 ($1.5\times 10^{5}$ selections in total) and aggregate the choices along each of the three state factors. Figure 20 reports the resulting selection frequencies, and three readable patterns emerge: First, crowding matters most: when an agent sits close to the current best (small $s_{J}$), the controller prefers the spherical surround search (about 31–34%), which refines locally, whereas a spread-out agent (large $s_{J}$) is far more often sent on the chaotic predation move (about 36–37%) that drives it toward the leader. Second, stagnation triggers a shift: an agent that has not improved for many iterations ($s_{\mathrm{stag}}\geq 15$) is moved away from the spiral search (down to 16%) and toward predation and compressed-space exploration (36% and 28%), i.e., the controller actively tries to dislodge stuck agents. Third, the mix evolves over the run: predation rises from 28% early on to 36% later while the more exploratory spiral and compressed-space moves recede, so the policy tilts gradually from exploration toward exploitation. These behaviors are learned online rather than hand-coded, and they mirror the intuition that motivated the state design.

### 6.6. Qualitative Analysis

The rankings in the previous subsections summarize *how well* CCO-RL performs, but they say little about *how* it searches. To make the behavior visible, we run the proposed algorithm in two dimensions, with the same population of 30 agents and 500 iterations, and record the full optimization history on every benchmark function. Two dimensions are used because the search history and the agent trajectory can only be drawn when the position vector has two components. All other settings match the main experiments. Each diagnostic figure collects six panels: the three-dimensional shape of the landscape, the search history (every position visited by every agent over the whole run, drawn over the function contours), the average fitness of the population per iteration, the trajectory of the first coordinate of a single agent, the best-so-far convergence curve on a logarithmic scale and the exploration versus exploitation balance measured from the population diversity.

Four patterns recur across the functions: First, the search history shows agents densely clustered around the global optimum with a thin scattering of points elsewhere, which confirms that the controller concentrates effort on the promising basin while still sampling the rest of the domain. Second, the trajectory of the first agent swings widely during the opening iterations and then settles into small oscillations, the textbook signature of a smooth transition from exploration to exploitation. Third, the average fitness and convergence curves both fall steeply in the first fifth of the run and then flatten, so most of the progress is made early and is refined thereafter. Fourth, the exploration–exploitation panel crosses over within roughly the first ten to twenty per cent of the iterations and is dominated by exploitation for the remainder, which is consistent with the learned policy reported in Section 6.5. The same behavior holds on the harder hybrid and composition functions, except that a handful of agents keep probing secondary regions for longer, which is the desirable response to a rugged, multi-funnel landscape.

For completeness, the diagnostic plots are reported for all 29 CEC2017 (D=2) functions (Figure 21, Figure 22, Figure 23, Figure 24, Figure 25, Figure 26, Figure 27, Figure 28, Figure 29, Figure 30, Figure 31, Figure 32, Figure 33, Figure 34, Figure 35, Figure 36, Figure 37, Figure 38, Figure 39, Figure 40, Figure 41, Figure 42, Figure 43, Figure 44, Figure 45, Figure 46, Figure 47, Figure 48 and Figure 49) and all 12 CEC2022 (D=2) functions (Figure 50, Figure 51, Figure 52, Figure 53, Figure 54, Figure 55, Figure 56, Figure 57, Figure 58, Figure 59, Figure 60 and Figure 61).

### 6.7. Scaling to D = 100

To probe scalability, we additionally compare CCO-RL with the base CCO on CEC2017 at D=100. As Table 8 shows, CCO-RL attains the lower mean on 26 of 29 functions, with a Wilcoxon Win/Tie/Loss record of 22/6/1, which confirms that the advantage of adaptive operator selection over the fixed CCO schedule not only persists but grows at the highest dimension. To be explicit about the trend that one reviewer raised: a larger *D* does *not* worsen (raise) CCO-RL’s rank; its CEC2017 Friedman rank remains the best and in fact improves slightly, from 1.62 at D=30 to 1.59 at D=50 (Table 6), and its head-to-head win rate over the base CCO climbs from 20/29 functions at D=30 to 26/29 at D=100. The harder, higher-dimensional landscapes are precisely where state-conditioned operator selection pays off most.

### 6.8. Runtime and Empirical Cost

Section 4.4 showed that CCO-RL is in the same asymptotic complexity class as the CCO. To make this concrete, and to compare the suites in practical terms, as suggested by a reviewer, Table 9 reports the mean wall-clock time per independent run measured on the common hardware. The Q-learning machinery adds only a small constant overhead. On CEC2017 D=30, CCO-RL averages 6.6 s per run against 5.4 s for plain CCO (about 23%), and the gap stays in this range at every dimension and for every suite. CCO-RL is comfortably mid-pack among the ten algorithms, faster than HHO, GA, DE, GWO and EO and within a small factor of the quickest methods, so its gain in solution quality does not come at the cost of a meaningful amount of time; runtime grows with dimension and with the cost of the objective itself, which is why every algorithm is slower on CEC2017 (D=50) than on the lower-dimensional CEC2022 (D=20).

## 7. Engineering Design Problems

**Figure 62:**
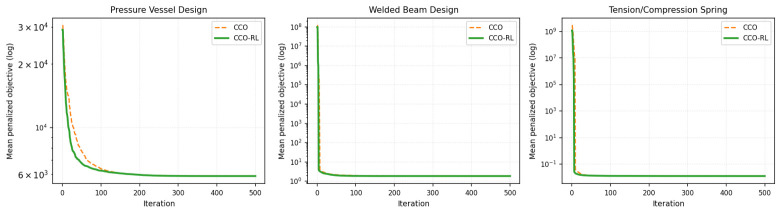
Mean convergence curves (mean penalized objective, log scale) on the three engineering design problems over 30 runs. CCO-RL (green, solid) versus CCO (orange, dashed).

To assess CCO-RL on real-world constrained optimization, we evaluate it against the base CCO on three classical mechanical engineering design problems widely used as metaheuristic benchmarks [31,32]: the pressure vessel design problem (four variables, four constraints) [33], the welded beam design problem (four variables, seven constraints) [34] and the tension/compression spring design problem (three variables, four constraints). Constraints are handled by the standard static penalty method [35]. Table 10 reports the mean penalized objective over 30 runs. CCO-RL attains the best mean design on all three problems and the lowest standard deviation on two, and pairwise Wilcoxon tests confirm significance at α=0.05 on every problem (pressure vessel $p=3.7\times 10^{-9}$; welded beam $p=3.2\times 10^{-11}$; spring $p=3.7\times 10^{-6}$). Figure 62 shows the convergence curves.

## 8. Discussion

The results tell a simple story: The CCO already comprises a good toolbox of moves; however, what holds it back is the blunt way it picks among them. Swap that fixed chain of coin flips for a controller that watches each agent and learns from experience, and the same toolbox leads in a strong field.

Pooled over the 70 instances, CCO-RL achieves the best Friedman rank (1.69) and is, according to the Nemenyi test, significantly ahead of all eight external competitors. It comes first on CEC2017 at both D=30 and D=50 and second on CEC2022, where the easier, lower-dimensional functions leave almost no daylight between it and the base CCO. The FGO is the toughest of the outsiders (rank 3.63), yet it still sits well outside the critical distance, and the classical methods trail far behind. Because CCO-RL and the CCO run the very same operators on the same budget, the whole gap between them, and CCO-RL’s lead overall, comes down to the controller rather than to any change in the moves themselves.

The controller keys its choice to three things: how crowded the agent is, how long it has gone without improving and how far the run has progressed. The ablation in Section 5.4 shows that the method is robust to this state design: removing any single feature leaves the Friedman rank statistically unchanged (p=0.19), while the full encoding stays among the best performers and needs no prior decision about which feature to drop. The advantage over the plain CCO is largest on the high-dimensional CEC2017 hybrids and compositions in the main comparison of Section 6, where picking the wrong move is costly and where noticing a stalled agent and switching it to exploration really matters. On the gentler CEC2022 functions, the margin over the plain CCO narrows. None of this hinges on careful tuning: across the swept ranges, the Friedman rank shifts by only 0.6 to 2.0, and the defaults land within half a rank of the best setting every time.

### Limitations

Several limitations should be stated plainly: First, the eight external competitors were evaluated on CEC2017 (D=30,50) and CEC2022 (D=20) but not at D=100 or on the engineering problems, so those two studies compare CCO-RL against the base CCO alone; a full multi-algorithm comparison at D=100 is left for future work. Second, the controller is deliberately lightweight: a single Q-table of 48 coarse states is shared by the whole swarm, which keeps learning fast and cheap but cannot specialize to an individual agent’s trajectory or capture finer gradations of crowding and progress. Third, the action set is fixed to the CCO’s four native moves; the controller can only choose among them and cannot invent or blend new operators. Fourth, like all tabular reinforcement learning, the method assumes that the chosen state discretization is informative. The ablation supports the present encoding, but a poorly chosen state could, in principle, slow learning. Finally, the study targets bound-constrained continuous optimization and three classical penalty-handled design problems, so extensions to genuinely constrained, multi-objective and discrete settings remain open.

## 9. Conclusions

We proposed CCO-RL, a Q-learning-enhanced variant of the Cuckoo Catfish Optimizer in which a single shared tabular Q-learning controller replaces the CCO’s fixed rand>C strategy cascade. The controller reads a 48-state picture of each agent (its crowding, its stagnation and the stage of the run), picks one of the CCO’s four moves with a decaying ε-greedy policy and learns online from a bounded improvement reward, without increasing the per-iteration function evaluation budget. Over 70 instances of CEC2017 (D=30,50) and CEC2022 (D=20), CCO-RL earned the best Friedman rank (1.69) of the ten algorithms, led both CEC2017 dimensions, came second on CEC2022 and was significantly better than all eight external competitors according to the Nemenyi test. It also returned the best mean design on three engineering problems and, in a D=100 check, beat the plain CCO on 26 of 29 functions. Future work will extend the external comparison to D=100 and to the engineering problems, replace the lookup table with a function approximator over continuous features for finer per-agent control and carry the idea over to constrained, multi-objective and discrete problems.

## Figures and Tables

**Figure 21:**
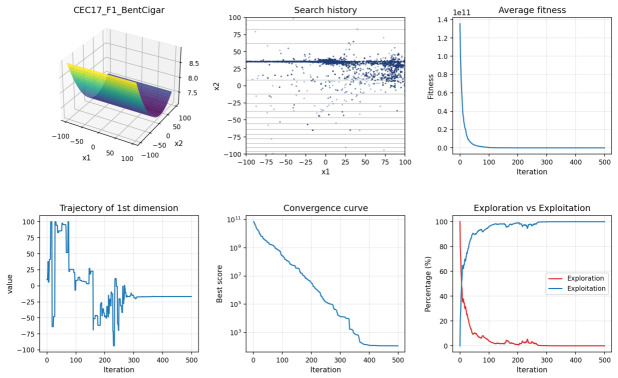
Qualitative analysis of CCO-RL on CEC2017 F1 (Bent Cigar): landscape, search history, average fitness, first-dimension trajectory, convergence curve and exploration–exploitation balance.

**Figure 22:**
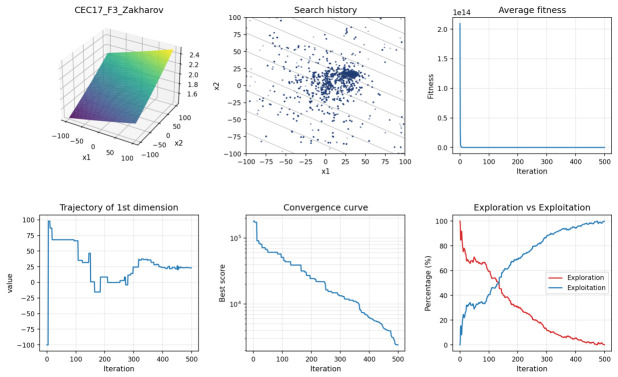
Qualitative analysis of CCO-RL on CEC2017 F3 (Zakharov): landscape, search history, average fitness, first-dimension trajectory, convergence curve and exploration–exploitation balance.

**Figure 23:**
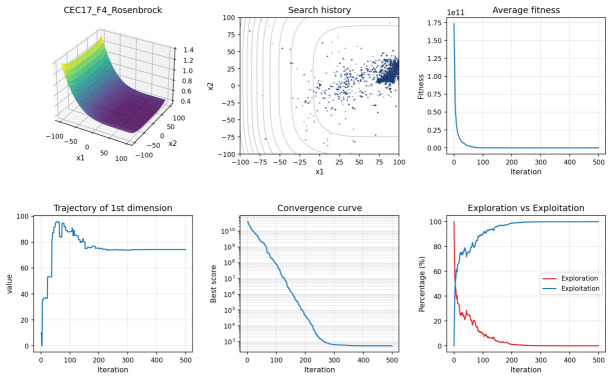
Qualitative analysis of CCO-RL on CEC2017 F4 (Rosenbrock): landscape, search history, average fitness, first-dimension trajectory, convergence curve and exploration–exploitation balance.

**Figure 24:**
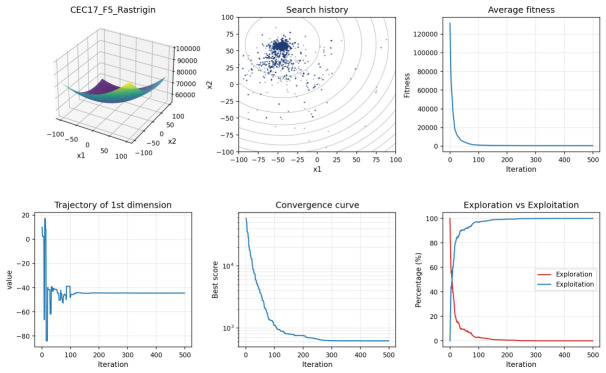
Qualitative analysis of CCO-RL on CEC2017 F5 (Rastrigin): landscape, search history, average fitness, first-dimension trajectory, convergence curve and exploration–exploitation balance.

**Figure 25:**
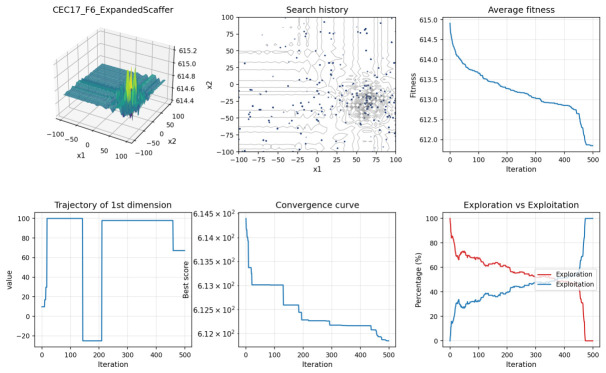
Qualitative analysis of CCO-RL on CEC2017 F6 (Expanded Scaffer): landscape, search history, average fitness, first-dimension trajectory, convergence curve and exploration–exploitation balance.

**Figure 26:**
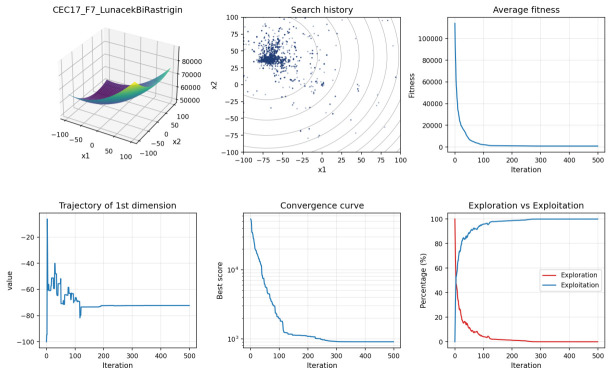
Qualitative analysis of CCO-RL on CEC2017 F7 (Lunacek Bi Rastrigin): landscape, search history, average fitness, first-dimension trajectory, convergence curve and exploration–exploitation balance.

**Figure 27:**
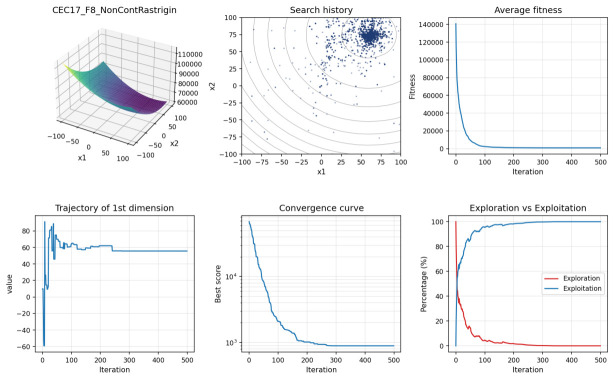
Qualitative analysis of CCO-RL on CEC2017 F8 (Non Cont Rastrigin): landscape, search history, average fitness, first-dimension trajectory, convergence curve and exploration–exploitation balance.

**Figure 28:**
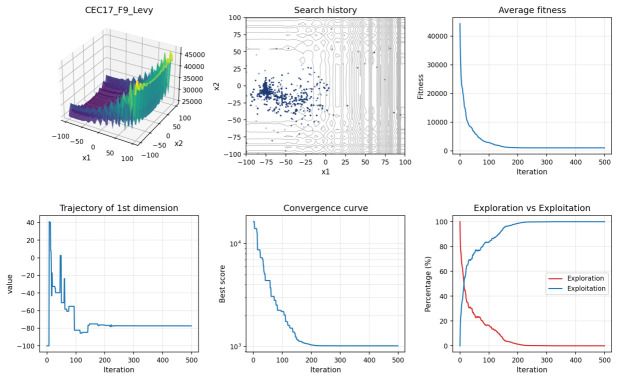
Qualitative analysis of CCO-RL on CEC2017 F9 (Levy): landscape, search history, average fitness, first-dimension trajectory, convergence curve and exploration–exploitation balance.

**Figure 29:**
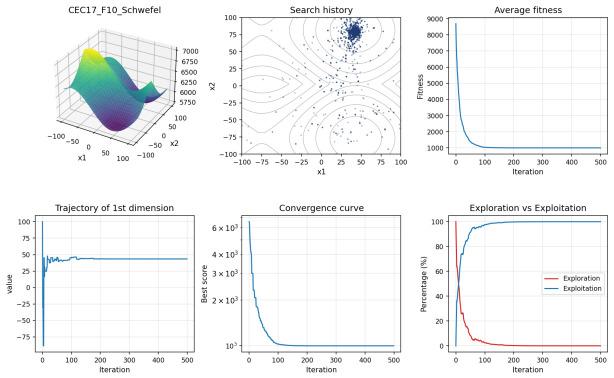
Qualitative analysis of CCO-RL on CEC2017 F10 (Schwefel): landscape, search history, average fitness, first-dimension trajectory, convergence curve and exploration–exploitation balance.

**Figure 30:**
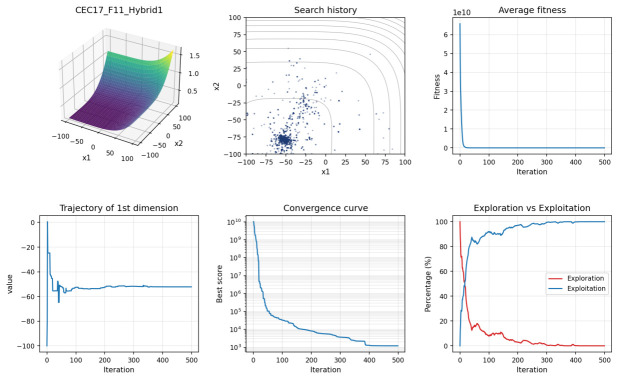
Qualitative analysis of CCO-RL on CEC2017 F11 (Hybrid1): landscape, search history, average fitness, first-dimension trajectory, convergence curve and exploration–exploitation balance.

**Figure 31:**
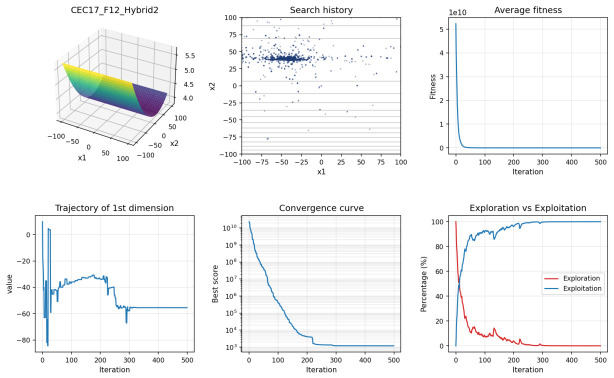
Qualitative analysis of CCO-RL on CEC2017 F12 (Hybrid2): landscape, search history, average fitness, first-dimension trajectory, convergence curve and exploration–exploitation balance.

**Figure 32:**
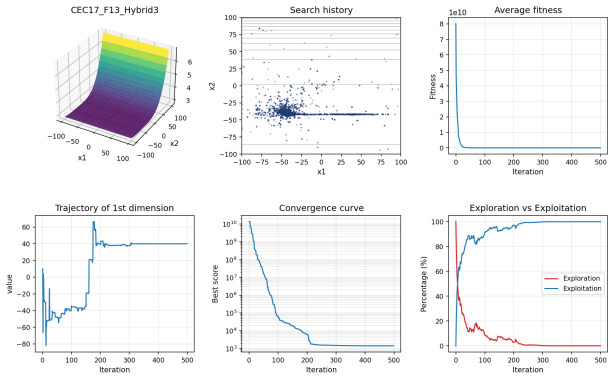
Qualitative analysis of CCO-RL on CEC2017 F13 (Hybrid3): landscape, search history, average fitness, first-dimension trajectory, convergence curve and exploration–exploitation balance.

**Figure 33:**
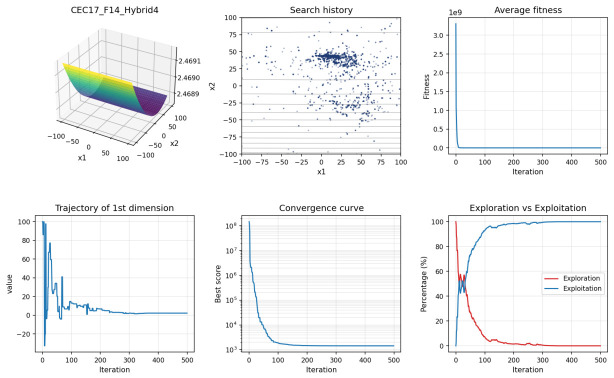
Qualitative analysis of CCO-RL on CEC2017 F14 (Hybrid4): landscape, search history, average fitness, first-dimension trajectory, convergence curve and exploration–exploitation balance.

**Figure 34:**
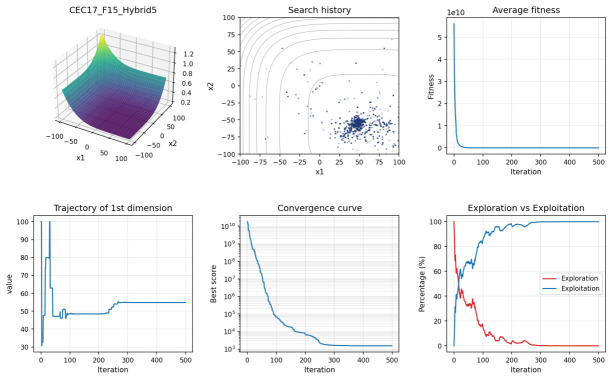
Qualitative analysis of CCO-RL on CEC2017 F15 (Hybrid5): landscape, search history, average fitness, first-dimension trajectory, convergence curve and exploration–exploitation balance.

**Figure 35:**
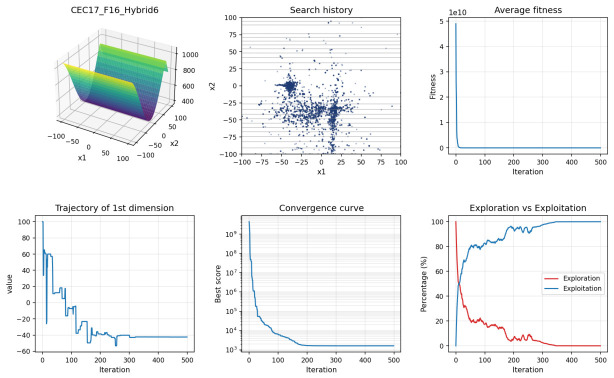
Qualitative analysis of CCO-RL on CEC2017 F16 (Hybrid6): landscape, search history, average fitness, first-dimension trajectory, convergence curve and exploration–exploitation balance.

**Figure 36:**
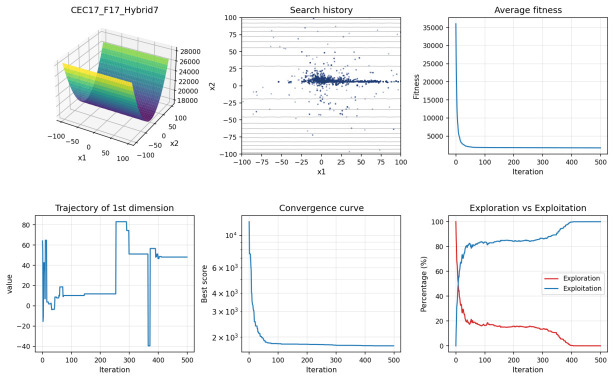
Qualitative analysis of CCO-RL on CEC2017 F17 (Hybrid7): landscape, search history, average fitness, first-dimension trajectory, convergence curve and exploration–exploitation balance.

**Figure 37:**
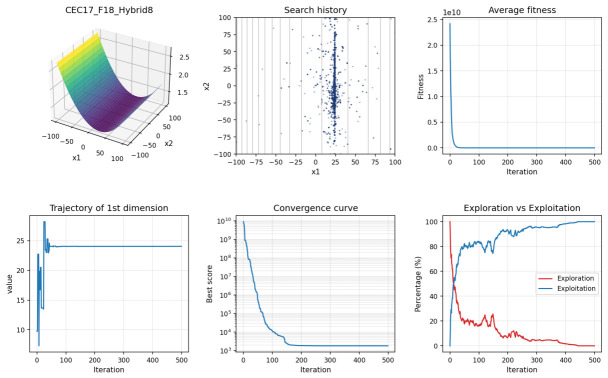
Qualitative analysis of CCO-RL on CEC2017 F18 (Hybrid8): landscape, search history, average fitness, first-dimension trajectory, convergence curve and exploration–exploitation balance.

**Figure 38:**
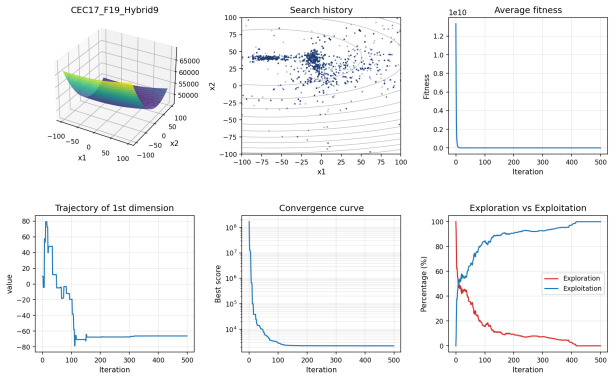
Qualitative analysis of CCO-RL on CEC2017 F19 (Hybrid9): landscape, search history, average fitness, first-dimension trajectory, convergence curve and exploration–exploitation balance.

**Figure 39:**
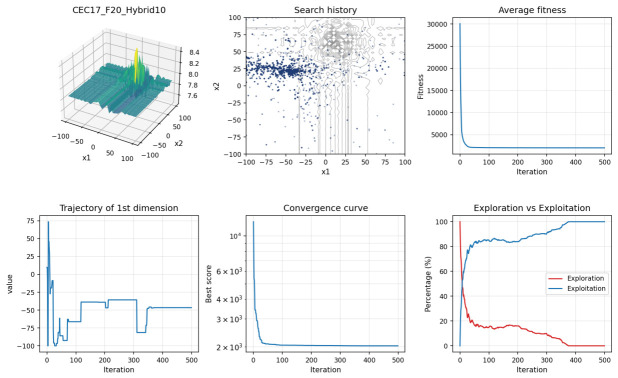
Qualitative analysis of CCO-RL on CEC2017 F20 (Hybrid10): landscape, search history, average fitness, first-dimension trajectory, convergence curve and exploration–exploitation balance.

**Figure 40:**
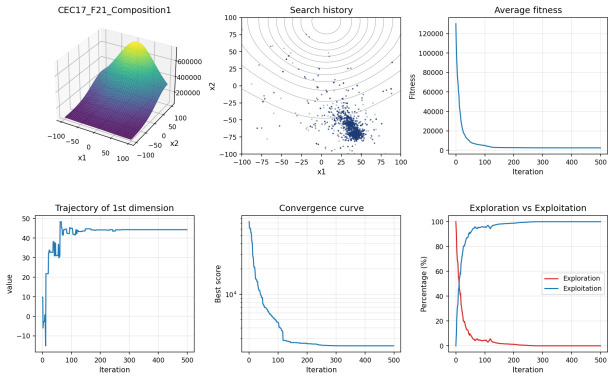
Qualitative analysis of CCO-RL on CEC2017 F21 (Composition1): landscape, search history, average fitness, first-dimension trajectory, convergence curve and exploration–exploitation balance.

**Figure 41:**
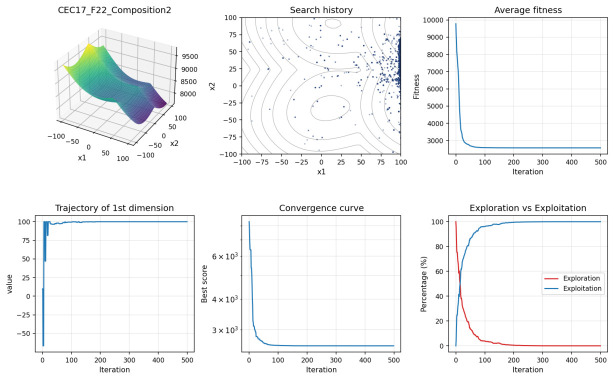
Qualitative analysis of CCO-RL on CEC2017 F22 (Composition2): landscape, search history, average fitness, first-dimension trajectory, convergence curve and exploration–exploitation balance.

**Figure 42:**
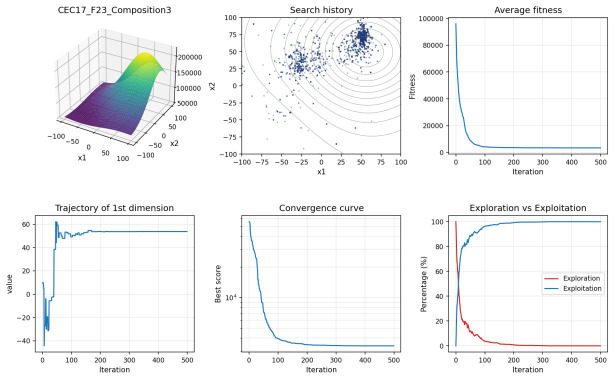
Qualitative analysis of CCO-RL on CEC2017 F23 (Composition3): landscape, search history, average fitness, first-dimension trajectory, convergence curve and exploration–exploitation balance.

**Figure 43:**
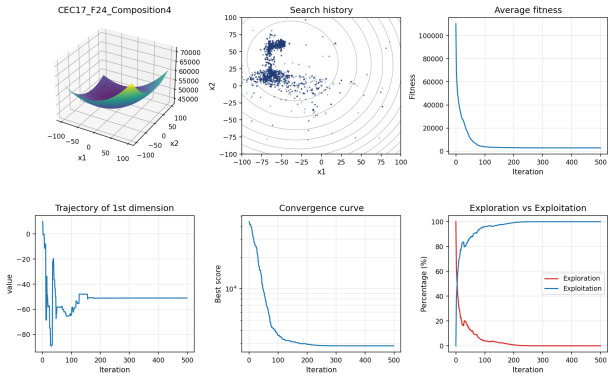
Qualitative analysis of CCO-RL on CEC2017 F24 (Composition4): landscape, search history, average fitness, first-dimension trajectory, convergence curve and exploration–exploitation balance.

**Figure 44:**
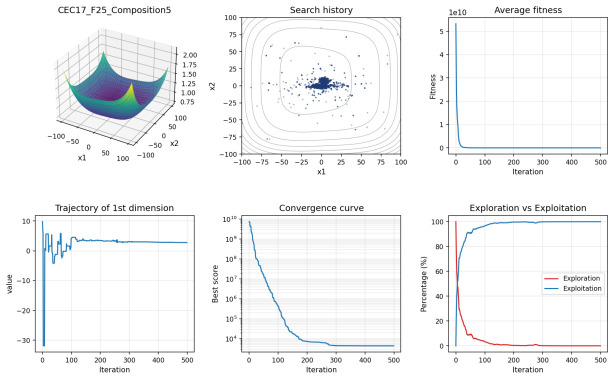
Qualitative analysis of CCO-RL on CEC2017 F25 (Composition5): landscape, search history, average fitness, first-dimension trajectory, convergence curve and exploration–exploitation balance.

**Figure 45:**
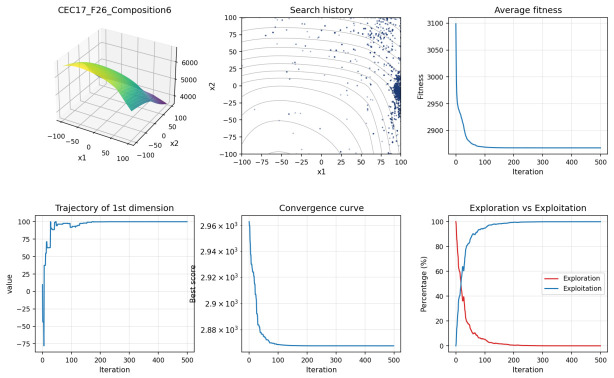
Qualitative analysis of CCO-RL on CEC2017 F26 (Composition6): landscape, search history, average fitness, first-dimension trajectory, convergence curve and exploration–exploitation balance.

**Figure 46:**
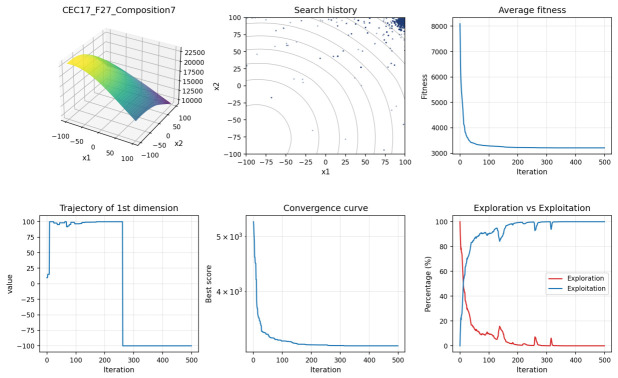
Qualitative analysis of CCO-RL on CEC2017 F27 (Composition7): landscape, search history, average fitness, first-dimension trajectory, convergence curve and exploration–exploitation balance.

**Figure 47:**
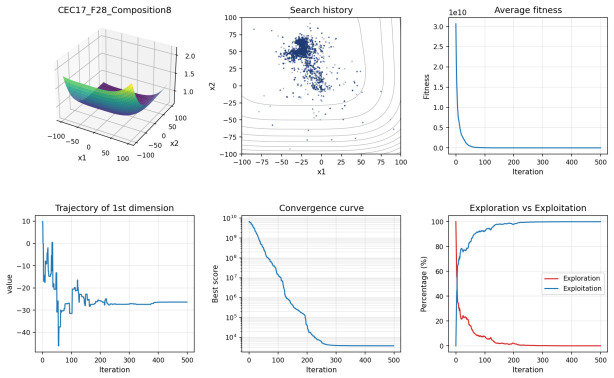
Qualitative analysis of CCO-RL on CEC2017 F28 (Composition8): landscape, search history, average fitness, first-dimension trajectory, convergence curve and exploration–exploitation balance.

**Figure 48:**
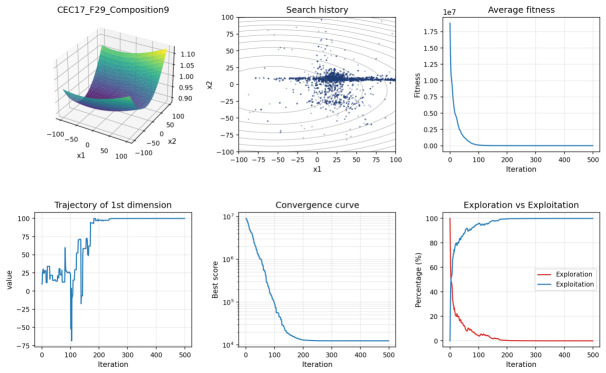
Qualitative analysis of CCO-RL on CEC2017 F29 (Composition9): landscape, search history, average fitness, first-dimension trajectory, convergence curve and exploration–exploitation balance.

**Figure 49:**
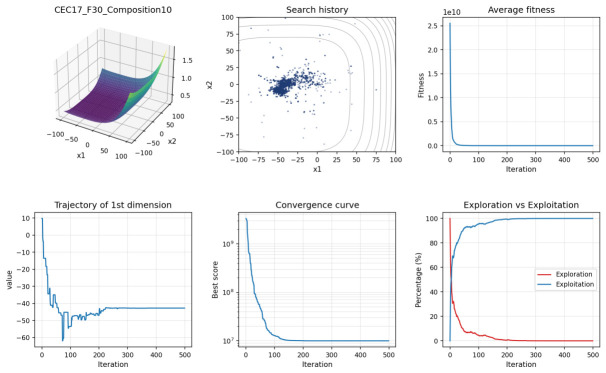
Qualitative analysis of CCO-RL on CEC2017 F30 (Composition10): landscape, search history, average fitness, first-dimension trajectory, convergence curve and exploration–exploitation balance.

**Figure 50:**
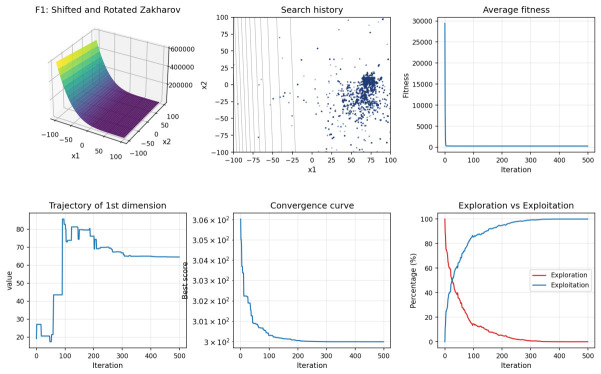
Qualitative analysis of CCO-RL on CEC2022 F1 (Shifted and Rotated Zakharov): landscape, search history, average fitness, first-dimension trajectory, convergence curve and exploration–exploitation balance.

**Figure 51:**
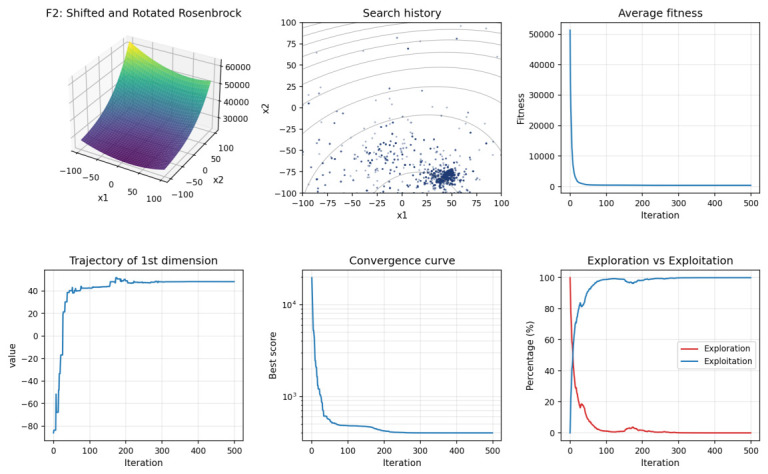
Qualitative analysis of CCO-RL on CEC2022 F2 (Shifted and Rotated Rosenbrock): landscape, search history, average fitness, first-dimension trajectory, convergence curve and exploration–exploitation balance.

**Figure 52:**
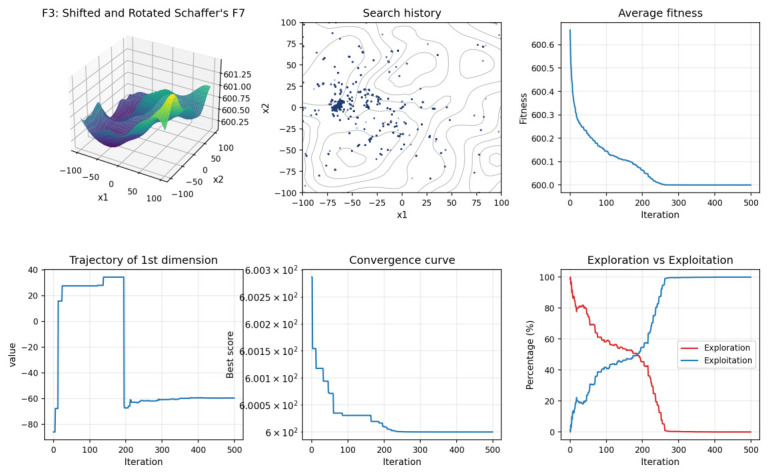
Qualitative analysis of CCO-RL on CEC2022 F3 (Shifted and Rotated Schaffer s F7): landscape, search history, average fitness, first-dimension trajectory, convergence curve and exploration–exploitation balance.

**Figure 53:**
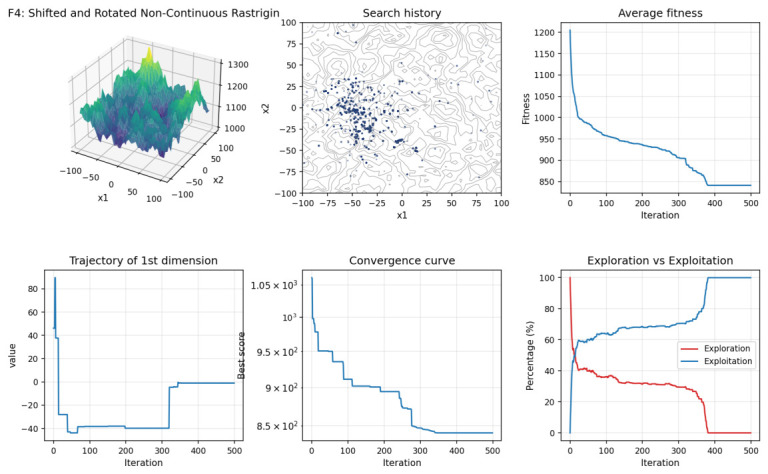
Qualitative analysis of CCO-RL on CEC2022 F4 (Shifted and Rotated Non Continuous Rastrigin): landscape, search history, average fitness, first-dimension trajectory, convergence curve and exploration–exploitation balance.

**Figure 54:**
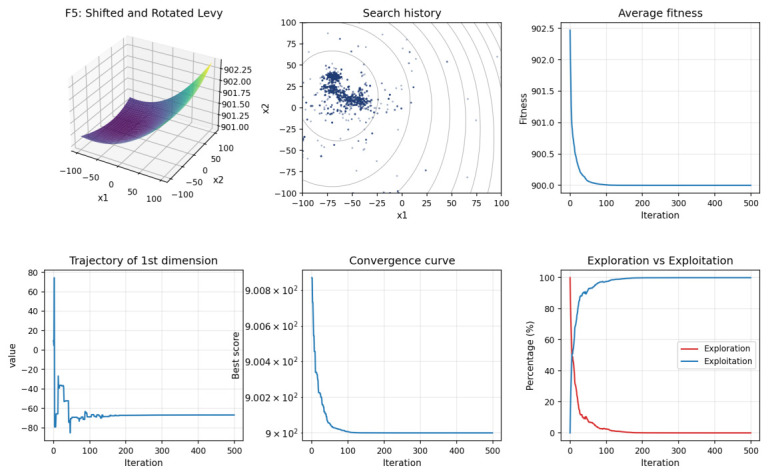
Qualitative analysis of CCO-RL on CEC2022 F5 (Shifted and Rotated Levy): landscape, search history, average fitness, first-dimension trajectory, convergence curve and exploration–exploitation balance.

**Figure 55:**
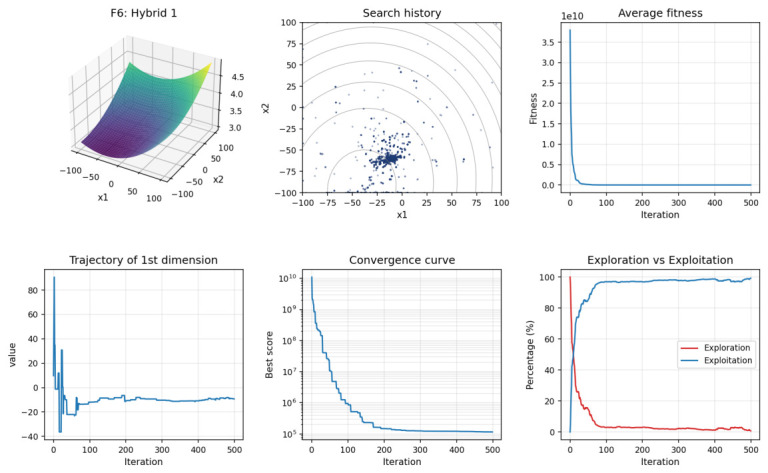
Qualitative analysis of CCO-RL on CEC2022 F6 (Hybrid 1 3 functions): landscape, search history, average fitness, first-dimension trajectory, convergence curve and exploration–exploitation balance.

**Figure 56:**
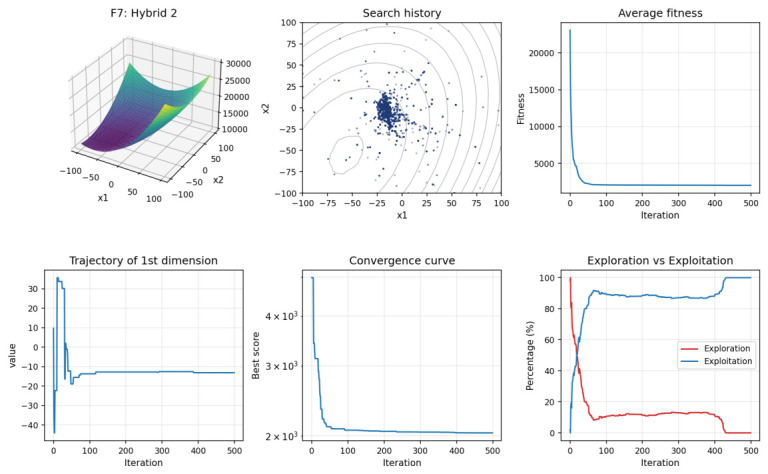
Qualitative analysis of CCO-RL on CEC2022 F7 (Hybrid 2 6 functions): landscape, search history, average fitness, first-dimension trajectory, convergence curve and exploration–exploitation balance.

**Figure 57:**
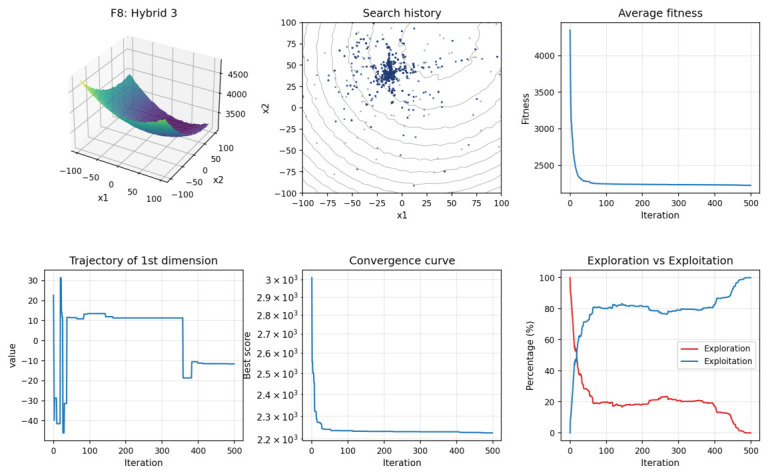
Qualitative analysis of CCO-RL on CEC2022 F8 (Hybrid 3 5 functions): landscape, search history, average fitness, first-dimension trajectory, convergence curve and exploration–exploitation balance.

**Figure 58:**
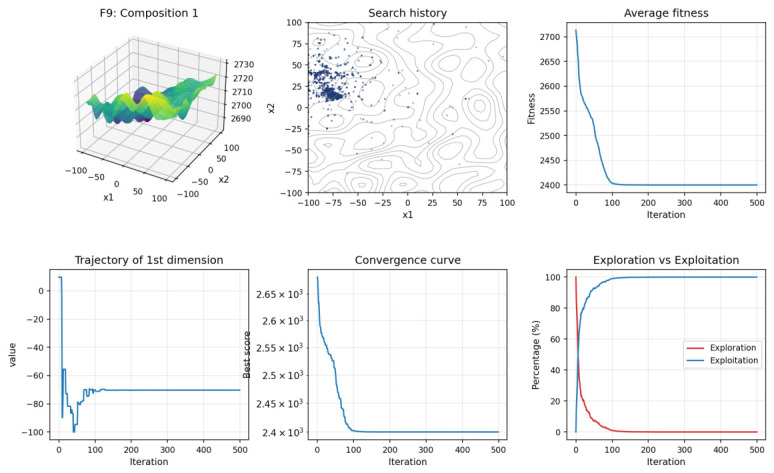
Qualitative analysis of CCO-RL on CEC2022 F9 (Composition 1 5 functions): landscape, search history, average fitness, first-dimension trajectory, convergence curve and exploration–exploitation balance.

**Figure 59:**
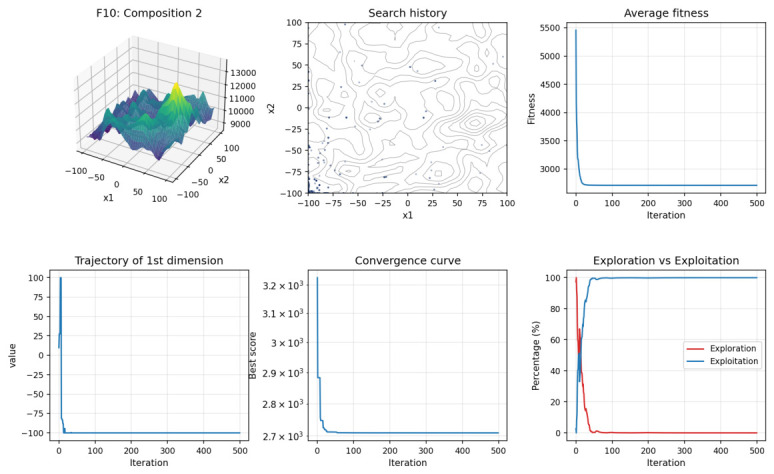
Qualitative analysis of CCO-RL on CEC2022 F10 (Composition 2 4 functions): landscape, search history, average fitness, first-dimension trajectory, convergence curve and exploration–exploitation balance.

**Figure 60:**
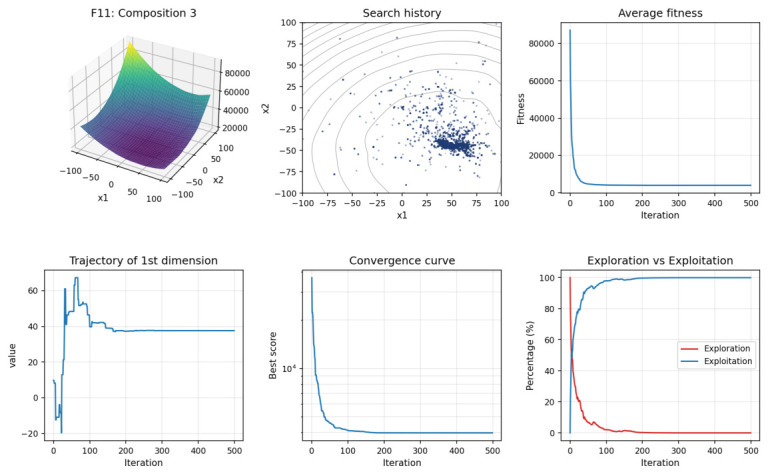
Qualitative analysis of CCO-RL on CEC2022 F11 (Composition 3 5 functions): landscape, search history, average fitness, first-dimension trajectory, convergence curve and exploration–exploitation balance.

**Figure 61:**
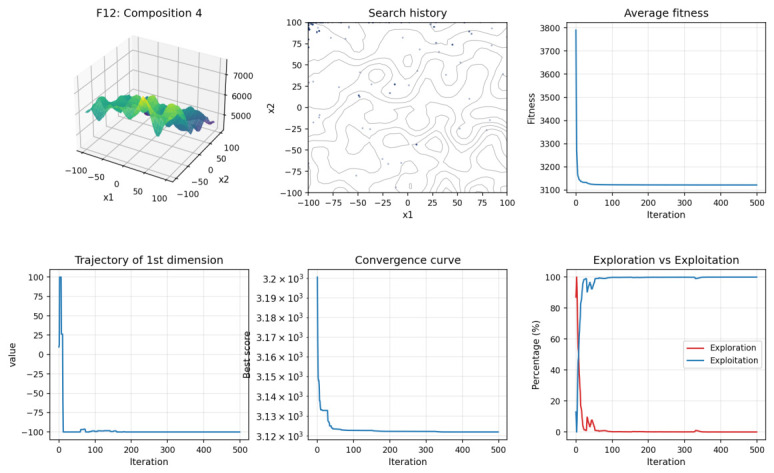
Qualitative analysis of CCO-RL on CEC2022 F12 (Composition 4 6 functions): landscape, search history, average fitness, first-dimension trajectory, convergence curve and exploration–exploitation balance.

**Table 1:** Parameter settings of the ten algorithms (common termination: N=30, T=500, 30 runs).

Algorithm	Parameters
GA [4]	crossover =0.8, mutation =0.1, tournament size =3
PSO [5]	w=0.7, $c_{1}=c_{2}=1.5$
DE [6]	F=0.5, CR=0.9 (DE/rand/1/bin)
GWO [7]	*a* linearly decreasing from 2 to 0
HHO [9]	β=1.5 (Lévy), $E_{0}\in \left[-1,1\right]$
RUN [11]	a=20, b=12, adaptive RK step
EO [10]	$a_{1}=2$, $a_{2}=1$, GP=0.5
FGO [12]	$E_{r}=0.8$, adaptive mycelium expansion
CCO [14]	p=0.9, death prob. $0.02\;T_{f}$, β=1.5 (Lévy)
CCO-RL (proposed)	α=0.1, γ=0.9, $\varepsilon_{\mathrm{start}}=1.0$, $\varepsilon_{\mathrm{end}}=0.1$

**Table 2:** Ablation study: mean best fitness (standard deviation in parentheses) of each CCO-RL variant over 30 runs on all 29 CEC2017 (D=30) functions. Best mean per row in bold. A lower Friedman rank is better.

Function	Full	NoQ	NoJi	NoStag	NoIter
F1_BentCigar	$1.21\;\times \;10^{2}$ ($6.16\;\times \;10^{1}$)	$2.99\;\times \;10^{2}$ ($7.65\;\times \;10^{2}$)	$1.99\;\times \;10^{2}$ ($2.55\;\times \;10^{2}$)	$1.81\;\times \;10^{2}$ ($3.78\;\times \;10^{2}$)	$5.11\;\times \;10^{2}$ ($1.54\;\times \;10^{3}$)
F3_Zakharov	$5.55\;\times \;10^{3}$ ($2.76\;\times \;10^{3}$)	$9.29\;\times \;10^{3}$ ($4.09\;\times \;10^{3}$)	$7.10\;\times \;10^{3}$ ($4.03\;\times \;10^{3}$)	$6.85\;\times \;10^{3}$ ($3.00\;\times \;10^{3}$)	$6.51\;\times \;10^{3}$ ($3.05\;\times \;10^{3}$)
F4_Rosenbrock	$5.45\;\times \;10^{2}$ ($1.90\;\times \;10^{2}$)	$5.47\;\times \;10^{2}$ ($1.49\;\times \;10^{2}$)	$5.02\;\times \;10^{2}$ ($1.11\;\times \;10^{2}$)	$5.21\;\times \;10^{2}$ ($8.62\;\times \;10^{1}$)	$5.63\;\times \;10^{2}$ ($1.60\;\times \;10^{2}$)
F5_Rastrigin	$6.44\;\times \;10^{2}$ ($8.08\;\times \;10^{1}$)	$6.44\;\times \;10^{2}$ ($1.24\;\times \;10^{2}$)	$6.28\;\times \;10^{2}$ ($7.15\;\times \;10^{1}$)	$6.59\;\times \;10^{2}$ ($1.64\;\times \;10^{2}$)	$6.37\;\times \;10^{2}$ ($8.30\;\times \;10^{1}$)
F6_ExpandedScaffer	$6.11\;\times \;10^{2}$ ($8.90\;\times \;10^{-1}$)	$6.11\;\times \;10^{2}$ ($7.40\;\times \;10^{-1}$)	$6.11\;\times \;10^{2}$ ($8.77\;\times \;10^{-1}$)	$6.11\;\times \;10^{2}$ ($8.66\;\times \;10^{-1}$)	$6.11\;\times \;10^{2}$ ($6.48\;\times \;10^{-1}$)
F7_LunacekBiRastrigin	$8.55\;\times \;10^{2}$ ($9.34\;\times \;10^{1}$)	$8.52\;\times \;10^{2}$ ($5.40\;\times \;10^{1}$)	$8.58\;\times \;10^{2}$ ($6.43\;\times \;10^{1}$)	$8.46\;\times \;10^{2}$ ($3.65\;\times \;10^{1}$)	$8.83\;\times \;10^{2}$ ($1.87\;\times \;10^{2}$)
F8_NonContRastrigin	$9.40\;\times \;10^{2}$ ($6.85\;\times \;10^{1}$)	$9.44\;\times \;10^{2}$ ($6.09\;\times \;10^{1}$)	$9.72\;\times \;10^{2}$ ($9.79\;\times \;10^{1}$)	$9.51\;\times \;10^{2}$ ($7.43\;\times \;10^{1}$)	$9.45\;\times \;10^{2}$ ($7.95\;\times \;10^{1}$)
F9_Levy	$1.21\;\times \;10^{3}$ ($3.33\;\times \;10^{2}$)	$1.22\;\times \;10^{3}$ ($3.55\;\times \;10^{2}$)	$1.12\;\times \;10^{3}$ ($1.74\;\times \;10^{2}$)	$1.13\;\times \;10^{3}$ ($2.63\;\times \;10^{2}$)	$1.22\;\times \;10^{3}$ ($3.67\;\times \;10^{2}$)
F10_Schwefel	$1.00\;\times \;10^{3}$ ($1.97\;\times \;10^{-2}$)	$1.00\;\times \;10^{3}$ ($3.11\;\times \;10^{-2}$)	$1.00\;\times \;10^{3}$ ($1.67\;\times \;10^{-2}$)	$1.00\;\times \;10^{3}$ ($1.89\;\times \;10^{-2}$)	$1.00\;\times \;10^{3}$ ($1.96\;\times \;10^{-2}$)
F11_Hybrid1	$1.97\;\times \;10^{3}$ ($1.15\;\times \;10^{3}$)	$2.00\;\times \;10^{3}$ ($6.93\;\times \;10^{2}$)	$2.07\;\times \;10^{3}$ ($8.66\;\times \;10^{2}$)	$1.58\;\times \;10^{3}$ ($4.20\;\times \;10^{2}$)	$2.10\;\times \;10^{3}$ ($1.68\;\times \;10^{3}$)
F12_Hybrid2	$1.20\;\times \;10^{3}$ ($9.58\;\times \;10^{0}$)	$1.21\;\times \;10^{3}$ ($2.17\;\times \;10^{1}$)	$1.20\;\times \;10^{3}$ ($7.77\;\times \;10^{0}$)	$1.20\;\times \;10^{3}$ ($2.39\;\times \;10^{0}$)	$1.22\;\times \;10^{3}$ ($6.69\;\times \;10^{1}$)
F13_Hybrid3	$1.51\;\times \;10^{3}$ ($2.21\;\times \;10^{2}$)	$1.66\;\times \;10^{3}$ ($5.44\;\times \;10^{2}$)	$1.49\;\times \;10^{3}$ ($1.98\;\times \;10^{2}$)	$1.44\;\times \;10^{3}$ ($2.19\;\times \;10^{2}$)	$1.49\;\times \;10^{3}$ ($1.55\;\times \;10^{2}$)
F14_Hybrid4	$1.44\;\times \;10^{3}$ ($2.49\;\times \;10^{1}$)	$1.44\;\times \;10^{3}$ ($1.25\;\times \;10^{1}$)	$1.44\;\times \;10^{3}$ ($1.75\;\times \;10^{1}$)	$1.44\;\times \;10^{3}$ ($1.25\;\times \;10^{1}$)	$1.44\;\times \;10^{3}$ ($1.81\;\times \;10^{1}$)
F15_Hybrid5	$1.76\;\times \;10^{3}$ ($3.18\;\times \;10^{2}$)	$1.74\;\times \;10^{3}$ ($2.76\;\times \;10^{2}$)	$1.98\;\times \;10^{3}$ ($1.34\;\times \;10^{3}$)	$1.92\;\times \;10^{3}$ ($6.38\;\times \;10^{2}$)	$1.75\;\times \;10^{3}$ ($3.88\;\times \;10^{2}$)
F16_Hybrid6	$1.73\;\times \;10^{3}$ ($1.81\;\times \;10^{2}$)	$1.68\;\times \;10^{3}$ ($1.26\;\times \;10^{2}$)	$1.75\;\times \;10^{3}$ ($2.72\;\times \;10^{2}$)	$1.74\;\times \;10^{3}$ ($2.30\;\times \;10^{2}$)	$1.72\;\times \;10^{3}$ ($1.82\;\times \;10^{2}$)
F17_Hybrid7	$1.74\;\times \;10^{3}$ ($6.98\;\times \;10^{0}$)	$1.74\;\times \;10^{3}$ ($6.94\;\times \;10^{0}$)	$1.74\;\times \;10^{3}$ ($9.73\;\times \;10^{0}$)	$1.74\;\times \;10^{3}$ ($5.62\;\times \;10^{0}$)	$1.74\;\times \;10^{3}$ ($7.08\;\times \;10^{0}$)
F18_Hybrid8	$1.84\;\times \;10^{3}$ ($4.28\;\times \;10^{1}$)	$1.84\;\times \;10^{3}$ ($1.26\;\times \;10^{1}$)	$1.86\;\times \;10^{3}$ ($1.10\;\times \;10^{2}$)	$1.84\;\times \;10^{3}$ ($3.18\;\times \;10^{1}$)	$1.85\;\times \;10^{3}$ ($4.39\;\times \;10^{1}$)
F19_Hybrid9	$1.98\;\times \;10^{3}$ ($8.64\;\times \;10^{1}$)	$1.96\;\times \;10^{3}$ ($5.15\;\times \;10^{1}$)	$1.96\;\times \;10^{3}$ ($1.13\;\times \;10^{2}$)	$1.99\;\times \;10^{3}$ ($1.51\;\times \;10^{2}$)	$2.01\;\times \;10^{3}$ ($1.75\;\times \;10^{2}$)
F20_Hybrid10	$2.03\;\times \;10^{3}$ ($2.98\;\times \;10^{0}$)	$2.03\;\times \;10^{3}$ ($4.34\;\times \;10^{0}$)	$2.03\;\times \;10^{3}$ ($4.63\;\times \;10^{0}$)	$2.03\;\times \;10^{3}$ ($5.15\;\times \;10^{0}$)	$2.03\;\times \;10^{3}$ ($4.68\;\times \;10^{0}$)
F21_Composition1	$3.15\;\times \;10^{3}$ ($1.91\;\times \;10^{3}$)	$2.99\;\times \;10^{3}$ ($1.58\;\times \;10^{3}$)	$3.14\;\times \;10^{3}$ ($1.91\;\times \;10^{3}$)	$2.98\;\times \;10^{3}$ ($1.58\;\times \;10^{3}$)	$3.15\;\times \;10^{3}$ ($1.91\;\times \;10^{3}$)
F22_Composition2	$2.53\;\times \;10^{3}$ ($7.99\;\times \;10^{1}$)	$2.53\;\times \;10^{3}$ ($7.69\;\times \;10^{1}$)	$2.54\;\times \;10^{3}$ ($7.30\;\times \;10^{1}$)	$2.54\;\times \;10^{3}$ ($7.37\;\times \;10^{1}$)	$2.52\;\times \;10^{3}$ ($7.94\;\times \;10^{1}$)
F23_Composition3	$4.13\;\times \;10^{3}$ ($4.06\;\times \;10^{3}$)	$3.36\;\times \;10^{3}$ ($3.31\;\times \;10^{1}$)	$4.90\;\times \;10^{3}$ ($5.64\;\times \;10^{3}$)	$3.41\;\times \;10^{3}$ ($1.38\;\times \;10^{2}$)	$4.12\;\times \;10^{3}$ ($4.06\;\times \;10^{3}$)
F24_Composition4	$2.83\;\times \;10^{3}$ ($3.87\;\times \;10^{1}$)	$2.83\;\times \;10^{3}$ ($4.16\;\times \;10^{1}$)	$2.86\;\times \;10^{3}$ ($1.25\;\times \;10^{2}$)	$3.55\;\times \;10^{3}$ ($3.74\;\times \;10^{3}$)	$2.83\;\times \;10^{3}$ ($3.92\;\times \;10^{1}$)
F25_Composition5	$4.43\;\times \;10^{3}$ ($5.51\;\times \;10^{1}$)	$4.44\;\times \;10^{3}$ ($7.24\;\times \;10^{1}$)	$4.42\;\times \;10^{3}$ ($3.05\;\times \;10^{1}$)	$4.42\;\times \;10^{3}$ ($8.49\;\times \;10^{1}$)	$4.44\;\times \;10^{3}$ ($5.99\;\times \;10^{1}$)
F26_Composition6	$2.88\;\times \;10^{3}$ ($2.02\;\times \;10^{1}$)	$2.88\;\times \;10^{3}$ ($2.29\;\times \;10^{1}$)	$2.87\;\times \;10^{3}$ ($1.84\;\times \;10^{1}$)	$2.88\;\times \;10^{3}$ ($2.17\;\times \;10^{1}$)	$2.88\;\times \;10^{3}$ ($2.02\;\times \;10^{1}$)
F27_Composition7	$3.22\;\times \;10^{3}$ ($1.20\;\times \;10^{1}$)	$3.22\;\times \;10^{3}$ ($1.74\;\times \;10^{1}$)	$3.22\;\times \;10^{3}$ ($1.03\;\times \;10^{1}$)	$3.22\;\times \;10^{3}$ ($4.65\;\times \;10^{0}$)	$3.22\;\times \;10^{3}$ ($1.23\;\times \;10^{1}$)
F28_Composition8	$3.71\;\times \;10^{3}$ ($5.56\;\times \;10^{1}$)	$3.71\;\times \;10^{3}$ ($3.81\;\times \;10^{1}$)	$3.72\;\times \;10^{3}$ ($4.87\;\times \;10^{1}$)	$3.71\;\times \;10^{3}$ ($4.13\;\times \;10^{1}$)	$3.73\;\times \;10^{3}$ ($6.29\;\times \;10^{1}$)
F29_Composition9	$1.25\;\times \;10^{4}$ ($1.09\;\times \;10^{-2}$)	$1.25\;\times \;10^{4}$ ($5.43\;\times \;10^{-2}$)	$1.25\;\times \;10^{4}$ ($2.33\;\times \;10^{-2}$)	$1.25\;\times \;10^{4}$ ($7.11\;\times \;10^{-3}$)	$1.25\;\times \;10^{4}$ ($1.43\;\times \;10^{-1}$)
F30_Composition10	$1.00\;\times \;10^{7}$ ($2.45\;\times \;10^{2}$)	$1.00\;\times \;10^{7}$ ($2.77\;\times \;10^{2}$)	$1.00\;\times \;10^{7}$ ($2.48\;\times \;10^{2}$)	$1.00\;\times \;10^{7}$ ($2.45\;\times \;10^{2}$)	$1.00\;\times \;10^{7}$ ($3.27\;\times \;10^{2}$)
**Friedman rank**	2.79	3.07	3.14	2.52	3.48

**Table 3:** CEC2017 (D=30) results: mean best fitness over 30 runs for all 10 algorithms. Best per row in bold. A lower Friedman rank is better.

Function	CCO-RL	CCO	GA	PSO	DE	GWO	HHO	RUN	EO	FGO
F1_BentCigar	$1.92\;\times \;10^{2}$	$6.01\;\times \;10^{3}$	$5.84\;\times \;10^{7}$	$2.03\;\times \;10^{10}$	$3.73\;\times \;10^{8}$	$2.25\;\times \;10^{9}$	$8.97\;\times \;10^{7}$	$1.04\;\times \;10^{10}$	$5.19\;\times \;10^{8}$	$1.99\;\times \;10^{5}$
F3_Zakharov	$6.64\;\times \;10^{3}$	$8.79\;\times \;10^{3}$	$1.92\;\times \;10^{4}$	$1.32\;\times \;10^{5}$	$1.01\;\times \;10^{5}$	$3.08\;\times \;10^{4}$	$1.27\;\times \;10^{5}$	$1.59\;\times \;10^{5}$	$1.17\;\times \;10^{5}$	$3.90\;\times \;10^{4}$
F4_Rosenbrock	$5.76\;\times \;10^{2}$	$7.66\;\times \;10^{2}$	$1.03\;\times \;10^{5}$	$7.68\;\times \;10^{9}$	$1.54\;\times \;10^{7}$	$1.82\;\times \;10^{8}$	$2.28\;\times \;10^{5}$	$3.03\;\times \;10^{9}$	$2.10\;\times \;10^{8}$	$3.35\;\times \;10^{4}$
F5_Rastrigin	$6.24\;\times \;10^{2}$	$5.95\;\times \;10^{2}$	$8.02\;\times \;10^{2}$	$1.86\;\times \;10^{4}$	$1.24\;\times \;10^{3}$	$2.93\;\times \;10^{3}$	$9.80\;\times \;10^{2}$	$1.15\;\times \;10^{4}$	$9.36\;\times \;10^{2}$	$6.39\;\times \;10^{2}$
F6_ExpandedScaffer	$6.11\;\times \;10^{2}$	$6.11\;\times \;10^{2}$	$6.10\;\times \;10^{2}$	$6.12\;\times \;10^{2}$	$6.13\;\times \;10^{2}$	$6.12\;\times \;10^{2}$	$6.13\;\times \;10^{2}$	$6.13\;\times \;10^{2}$	$6.12\;\times \;10^{2}$	$6.12\;\times \;10^{2}$
F7_LunacekBiRastrigin	$8.54\;\times \;10^{2}$	$8.17\;\times \;10^{2}$	$1.01\;\times \;10^{3}$	$1.88\;\times \;10^{4}$	$1.40\;\times \;10^{3}$	$2.46\;\times \;10^{3}$	$1.21\;\times \;10^{3}$	$9.71\;\times \;10^{3}$	$1.37\;\times \;10^{3}$	$8.73\;\times \;10^{2}$
F8_NonContRastrigin	$9.58\;\times \;10^{2}$	$9.06\;\times \;10^{2}$	$1.08\;\times \;10^{3}$	$2.09\;\times \;10^{4}$	$1.57\;\times \;10^{3}$	$2.84\;\times \;10^{3}$	$1.35\;\times \;10^{3}$	$1.08\;\times \;10^{4}$	$1.93\;\times \;10^{3}$	$9.33\;\times \;10^{2}$
F9_Levy	$1.16\;\times \;10^{3}$	$9.58\;\times \;10^{2}$	$4.41\;\times \;10^{3}$	$5.55\;\times \;10^{3}$	$1.77\;\times \;10^{3}$	$1.71\;\times \;10^{3}$	$8.29\;\times \;10^{3}$	$6.00\;\times \;10^{3}$	$2.18\;\times \;10^{3}$	$1.01\;\times \;10^{3}$
F10_Schwefel	$1.00\;\times \;10^{3}$	$1.00\;\times \;10^{3}$	$1.31\;\times \;10^{3}$	$2.32\;\times \;10^{3}$	$1.20\;\times \;10^{3}$	$1.23\;\times \;10^{3}$	$1.18\;\times \;10^{3}$	$2.08\;\times \;10^{3}$	$1.15\;\times \;10^{3}$	$1.09\;\times \;10^{3}$
F11_Hybrid1	$2.00\;\times \;10^{3}$	$3.90\;\times \;10^{3}$	$3.02\;\times \;10^{4}$	$4.51\;\times \;10^{8}$	$6.88\;\times \;10^{4}$	$1.18\;\times \;10^{6}$	$1.02\;\times \;10^{5}$	$9.16\;\times \;10^{6}$	$3.64\;\times \;10^{6}$	$8.17\;\times \;10^{4}$
F12_Hybrid2	$1.22\;\times \;10^{3}$	$1.83\;\times \;10^{3}$	$5.76\;\times \;10^{5}$	$4.75\;\times \;10^{9}$	$6.83\;\times \;10^{5}$	$1.45\;\times \;10^{8}$	$1.03\;\times \;10^{8}$	$7.11\;\times \;10^{8}$	$2.97\;\times \;10^{8}$	$2.57\;\times \;10^{7}$
F13_Hybrid3	$1.57\;\times \;10^{3}$	$2.36\;\times \;10^{3}$	$8.84\;\times \;10^{4}$	$3.09\;\times \;10^{9}$	$3.32\;\times \;10^{5}$	$1.43\;\times \;10^{8}$	$1.44\;\times \;10^{7}$	$7.79\;\times \;10^{8}$	$2.17\;\times \;10^{7}$	$2.13\;\times \;10^{6}$
F14_Hybrid4	$1.44\;\times \;10^{3}$	$1.44\;\times \;10^{3}$	$2.00\;\times \;10^{3}$	$8.49\;\times \;10^{5}$	$2.09\;\times \;10^{3}$	$1.16\;\times \;10^{5}$	$3.13\;\times \;10^{5}$	$3.82\;\times \;10^{5}$	$1.05\;\times \;10^{4}$	$1.21\;\times \;10^{5}$
F15_Hybrid5	$1.93\;\times \;10^{3}$	$3.29\;\times \;10^{3}$	$3.59\;\times \;10^{4}$	$1.74\;\times \;10^{9}$	$4.44\;\times \;10^{4}$	$6.35\;\times \;10^{7}$	$3.41\;\times \;10^{6}$	$1.44\;\times \;10^{8}$	$1.97\;\times \;10^{6}$	$4.40\;\times \;10^{5}$
F16_Hybrid6	$1.71\;\times \;10^{3}$	$1.69\;\times \;10^{3}$	$3.08\;\times \;10^{3}$	$1.75\;\times \;10^{8}$	$2.27\;\times \;10^{3}$	$2.50\;\times \;10^{4}$	$3.92\;\times \;10^{4}$	$3.89\;\times \;10^{6}$	$1.42\;\times \;10^{4}$	$3.40\;\times \;10^{4}$
F17_Hybrid7	$1.74\;\times \;10^{3}$	$1.74\;\times \;10^{3}$	$1.96\;\times \;10^{3}$	$3.16\;\times \;10^{3}$	$1.82\;\times \;10^{3}$	$1.97\;\times \;10^{3}$	$2.22\;\times \;10^{3}$	$2.45\;\times \;10^{3}$	$1.87\;\times \;10^{3}$	$1.88\;\times \;10^{3}$
F18_Hybrid8	$1.83\;\times \;10^{3}$	$1.88\;\times \;10^{3}$	$7.81\;\times \;10^{3}$	$2.88\;\times \;10^{8}$	$3.77\;\times \;10^{3}$	$9.42\;\times \;10^{6}$	$9.93\;\times \;10^{6}$	$2.87\;\times \;10^{7}$	$1.66\;\times \;10^{5}$	$3.94\;\times \;10^{6}$
F19_Hybrid9	$1.95\;\times \;10^{3}$	$1.94\;\times \;10^{3}$	$2.25\;\times \;10^{3}$	$5.82\;\times \;10^{4}$	$2.11\;\times \;10^{3}$	$2.81\;\times \;10^{4}$	$2.13\;\times \;10^{4}$	$6.17\;\times \;10^{4}$	$5.31\;\times \;10^{3}$	$1.63\;\times \;10^{4}$
F20_Hybrid10	$2.03\;\times \;10^{3}$	$2.03\;\times \;10^{3}$	$2.14\;\times \;10^{3}$	$3.24\;\times \;10^{3}$	$2.08\;\times \;10^{3}$	$2.21\;\times \;10^{3}$	$2.30\;\times \;10^{3}$	$2.42\;\times \;10^{3}$	$2.21\;\times \;10^{3}$	$2.11\;\times \;10^{3}$
F21_Composition1	$2.55\;\times \;10^{3}$	$2.70\;\times \;10^{3}$	$4.12\;\times \;10^{3}$	$2.05\;\times \;10^{4}$	$5.32\;\times \;10^{3}$	$4.85\;\times \;10^{3}$	$3.08\;\times \;10^{3}$	$1.41\;\times \;10^{4}$	$3.37\;\times \;10^{3}$	$3.17\;\times \;10^{3}$
F22_Composition2	$2.54\;\times \;10^{3}$	$2.58\;\times \;10^{3}$	$2.86\;\times \;10^{3}$	$3.36\;\times \;10^{3}$	$2.59\;\times \;10^{3}$	$2.67\;\times \;10^{3}$	$2.71\;\times \;10^{3}$	$2.68\;\times \;10^{3}$	$2.64\;\times \;10^{3}$	$2.61\;\times \;10^{3}$
F23_Composition3	$4.89\;\times \;10^{3}$	$3.36\;\times \;10^{3}$	$1.33\;\times \;10^{4}$	$2.09\;\times \;10^{4}$	$2.33\;\times \;10^{4}$	$8.43\;\times \;10^{3}$	$4.46\;\times \;10^{3}$	$2.52\;\times \;10^{4}$	$1.26\;\times \;10^{4}$	$6.41\;\times \;10^{3}$
F24_Composition4	$2.87\;\times \;10^{3}$	$3.51\;\times \;10^{3}$	$8.54\;\times \;10^{3}$	$2.59\;\times \;10^{4}$	$2.31\;\times \;10^{4}$	$1.20\;\times \;10^{4}$	$3.86\;\times \;10^{3}$	$2.03\;\times \;10^{4}$	$1.49\;\times \;10^{4}$	$9.12\;\times \;10^{3}$
F25_Composition5	$4.42\;\times \;10^{3}$	$4.54\;\times \;10^{3}$	$7.21\;\times \;10^{4}$	$2.93\;\times \;10^{9}$	$1.04\;\times \;10^{7}$	$3.87\;\times \;10^{7}$	$2.66\;\times \;10^{5}$	$1.17\;\times \;10^{9}$	$1.26\;\times \;10^{7}$	$3.46\;\times \;10^{4}$
F26_Composition6	$2.88\;\times \;10^{3}$	$2.87\;\times \;10^{3}$	$2.87\;\times \;10^{3}$	$2.93\;\times \;10^{3}$	$2.88\;\times \;10^{3}$	$2.87\;\times \;10^{3}$	$2.87\;\times \;10^{3}$	$2.92\;\times \;10^{3}$	$2.90\;\times \;10^{3}$	$2.87\;\times \;10^{3}$
F27_Composition7	$3.22\;\times \;10^{3}$	$3.23\;\times \;10^{3}$	$3.27\;\times \;10^{3}$	$3.51\;\times \;10^{3}$	$3.26\;\times \;10^{3}$	$3.39\;\times \;10^{3}$	$3.55\;\times \;10^{3}$	$3.34\;\times \;10^{3}$	$3.31\;\times \;10^{3}$	$3.33\;\times \;10^{3}$
F28_Composition8	$3.72\;\times \;10^{3}$	$3.79\;\times \;10^{3}$	$4.90\;\times \;10^{8}$	$2.85\;\times \;10^{9}$	$4.62\;\times \;10^{9}$	$2.90\;\times \;10^{8}$	$2.86\;\times \;10^{8}$	$4.70\;\times \;10^{9}$	$2.80\;\times \;10^{9}$	$4.11\;\times \;10^{4}$
F29_Composition9	$1.25\;\times \;10^{4}$	$1.25\;\times \;10^{4}$	$2.76\;\times \;10^{4}$	$2.94\;\times \;10^{6}$	$9.11\;\times \;10^{4}$	$6.54\;\times \;10^{5}$	$3.23\;\times \;10^{4}$	$3.64\;\times \;10^{6}$	$6.05\;\times \;10^{4}$	$1.42\;\times \;10^{4}$
F30_Composition10	$1.00\;\times \;10^{7}$	$1.00\;\times \;10^{7}$	$1.02\;\times \;10^{7}$	$1.46\;\times \;10^{9}$	$1.43\;\times \;10^{7}$	$6.44\;\times \;10^{7}$	$1.03\;\times \;10^{7}$	$6.96\;\times \;10^{8}$	$6.45\;\times \;10^{7}$	$1.01\;\times \;10^{7}$
**Friedman rank**	1.62	1.76	4.45	9.55	5.48	6.72	6.10	9.03	6.17	4.10

**Table 4:** CEC2017 (D=50) results: mean best fitness over 30 runs for all 10 algorithms. Best per row in bold. A lower Friedman rank is better.

Function	CCO-RL	CCO	GA	PSO	DE	GWO	HHO	RUN	EO	FGO
F1_BentCigar	$9.13\;\times \;10^{4}$	$2.08\;\times \;10^{6}$	$6.40\;\times \;10^{8}$	$6.20\;\times \;10^{10}$	$4.17\;\times \;10^{9}$	$8.71\;\times \;10^{9}$	$3.71\;\times \;10^{8}$	$3.56\;\times \;10^{10}$	$9.71\;\times \;10^{8}$	$1.81\;\times \;10^{6}$
F3_Zakharov	$4.22\;\times \;10^{4}$	$5.15\;\times \;10^{4}$	$8.01\;\times \;10^{4}$	$2.83\;\times \;10^{5}$	$2.31\;\times \;10^{5}$	$6.51\;\times \;10^{4}$	$2.11\;\times \;10^{5}$	$3.25\;\times \;10^{5}$	$2.63\;\times \;10^{5}$	$1.16\;\times \;10^{5}$
F4_Rosenbrock	$3.44\;\times \;10^{3}$	$1.36\;\times \;10^{4}$	$3.68\;\times \;10^{6}$	$3.20\;\times \;10^{10}$	$4.42\;\times \;10^{8}$	$7.26\;\times \;10^{8}$	$1.71\;\times \;10^{6}$	$1.27\;\times \;10^{10}$	$3.77\;\times \;10^{8}$	$1.15\;\times \;10^{5}$
F5_Rastrigin	$1.24\;\times \;10^{3}$	$1.23\;\times \;10^{3}$	$1.72\;\times \;10^{3}$	$6.24\;\times \;10^{4}$	$5.94\;\times \;10^{3}$	$9.32\;\times \;10^{3}$	$1.69\;\times \;10^{3}$	$3.65\;\times \;10^{4}$	$2.55\;\times \;10^{3}$	$8.39\;\times \;10^{2}$
F6_ExpandedScaffer	$6.20\;\times \;10^{2}$	$6.21\;\times \;10^{2}$	$6.20\;\times \;10^{2}$	$6.22\;\times \;10^{2}$	$6.23\;\times \;10^{2}$	$6.21\;\times \;10^{2}$	$6.22\;\times \;10^{2}$	$6.23\;\times \;10^{2}$	$6.22\;\times \;10^{2}$	$6.21\;\times \;10^{2}$
F7_LunacekBiRastrigin	$1.43\;\times \;10^{3}$	$1.47\;\times \;10^{3}$	$1.84\;\times \;10^{3}$	$4.38\;\times \;10^{4}$	$6.47\;\times \;10^{3}$	$7.87\;\times \;10^{3}$	$1.86\;\times \;10^{3}$	$2.83\;\times \;10^{4}$	$3.23\;\times \;10^{3}$	$1.09\;\times \;10^{3}$
F8_NonContRastrigin	$1.90\;\times \;10^{3}$	$1.60\;\times \;10^{3}$	$1.94\;\times \;10^{3}$	$5.51\;\times \;10^{4}$	$6.43\;\times \;10^{3}$	$8.40\;\times \;10^{3}$	$2.13\;\times \;10^{3}$	$3.53\;\times \;10^{4}$	$3.74\;\times \;10^{3}$	$1.14\;\times \;10^{3}$
F9_Levy	$2.74\;\times \;10^{3}$	$1.89\;\times \;10^{3}$	$1.32\;\times \;10^{4}$	$1.79\;\times \;10^{4}$	$6.35\;\times \;10^{3}$	$4.59\;\times \;10^{3}$	$1.56\;\times \;10^{4}$	$2.18\;\times \;10^{4}$	$4.21\;\times \;10^{3}$	$1.79\;\times \;10^{3}$
F10_Schwefel	$1.00\;\times \;10^{3}$	$1.00\;\times \;10^{3}$	$2.05\;\times \;10^{3}$	$4.95\;\times \;10^{3}$	$2.30\;\times \;10^{3}$	$1.87\;\times \;10^{3}$	$1.33\;\times \;10^{3}$	$4.31\;\times \;10^{3}$	$1.68\;\times \;10^{3}$	$1.20\;\times \;10^{3}$
F11_Hybrid1	$2.03\;\times \;10^{4}$	$4.06\;\times \;10^{4}$	$1.75\;\times \;10^{5}$	$3.37\;\times \;10^{9}$	$5.83\;\times \;10^{6}$	$1.39\;\times \;10^{8}$	$2.24\;\times \;10^{5}$	$1.04\;\times \;10^{9}$	$3.49\;\times \;10^{7}$	$1.59\;\times \;10^{5}$
F12_Hybrid2	$1.01\;\times \;10^{4}$	$6.91\;\times \;10^{4}$	$2.85\;\times \;10^{7}$	$8.36\;\times \;10^{9}$	$9.65\;\times \;10^{7}$	$1.03\;\times \;10^{9}$	$2.00\;\times \;10^{8}$	$4.21\;\times \;10^{9}$	$1.01\;\times \;10^{8}$	$7.59\;\times \;10^{7}$
F13_Hybrid3	$6.91\;\times \;10^{3}$	$7.53\;\times \;10^{4}$	$5.54\;\times \;10^{6}$	$1.29\;\times \;10^{10}$	$8.06\;\times \;10^{7}$	$1.17\;\times \;10^{9}$	$6.26\;\times \;10^{7}$	$6.80\;\times \;10^{9}$	$3.41\;\times \;10^{8}$	$8.15\;\times \;10^{6}$
F14_Hybrid4	$1.55\;\times \;10^{3}$	$1.74\;\times \;10^{3}$	$3.73\;\times \;10^{3}$	$6.61\;\times \;10^{6}$	$4.63\;\times \;10^{3}$	$2.77\;\times \;10^{6}$	$1.15\;\times \;10^{6}$	$2.30\;\times \;10^{6}$	$2.18\;\times \;10^{5}$	$7.03\;\times \;10^{5}$
F15_Hybrid5	$6.66\;\times \;10^{3}$	$3.08\;\times \;10^{4}$	$5.60\;\times \;10^{5}$	$6.73\;\times \;10^{9}$	$1.09\;\times \;10^{7}$	$3.48\;\times \;10^{8}$	$2.84\;\times \;10^{7}$	$1.05\;\times \;10^{9}$	$4.76\;\times \;10^{7}$	$3.41\;\times \;10^{6}$
F16_Hybrid6	$1.65\;\times \;10^{3}$	$1.77\;\times \;10^{3}$	$6.94\;\times \;10^{3}$	$7.05\;\times \;10^{8}$	$1.35\;\times \;10^{4}$	$4.94\;\times \;10^{6}$	$2.20\;\times \;10^{5}$	$1.18\;\times \;10^{8}$	$2.44\;\times \;10^{7}$	$1.16\;\times \;10^{5}$
F17_Hybrid7	$1.76\;\times \;10^{3}$	$1.76\;\times \;10^{3}$	$2.17\;\times \;10^{3}$	$9.03\;\times \;10^{3}$	$2.03\;\times \;10^{3}$	$3.05\;\times \;10^{3}$	$3.30\;\times \;10^{3}$	$4.75\;\times \;10^{3}$	$2.54\;\times \;10^{3}$	$2.17\;\times \;10^{3}$
F18_Hybrid8	$2.19\;\times \;10^{3}$	$5.99\;\times \;10^{3}$	$2.60\;\times \;10^{5}$	$2.47\;\times \;10^{9}$	$2.63\;\times \;10^{5}$	$5.28\;\times \;10^{7}$	$5.80\;\times \;10^{7}$	$5.99\;\times \;10^{8}$	$1.51\;\times \;10^{8}$	$1.56\;\times \;10^{7}$
F19_Hybrid9	$2.10\;\times \;10^{3}$	$2.12\;\times \;10^{3}$	$3.46\;\times \;10^{3}$	$1.56\;\times \;10^{5}$	$3.62\;\times \;10^{3}$	$3.09\;\times \;10^{4}$	$3.30\;\times \;10^{4}$	$1.76\;\times \;10^{5}$	$1.31\;\times \;10^{4}$	$2.90\;\times \;10^{4}$
F20_Hybrid10	$2.05\;\times \;10^{3}$	$2.04\;\times \;10^{3}$	$2.40\;\times \;10^{3}$	$6.61\;\times \;10^{3}$	$2.22\;\times \;10^{3}$	$2.86\;\times \;10^{3}$	$2.85\;\times \;10^{3}$	$4.17\;\times \;10^{3}$	$3.04\;\times \;10^{3}$	$2.26\;\times \;10^{3}$
F21_Composition1	$4.05\;\times \;10^{3}$	$4.65\;\times \;10^{3}$	$4.35\;\times \;10^{3}$	$5.72\;\times \;10^{4}$	$8.30\;\times \;10^{3}$	$1.16\;\times \;10^{4}$	$3.57\;\times \;10^{3}$	$3.96\;\times \;10^{4}$	$4.36\;\times \;10^{3}$	$3.52\;\times \;10^{3}$
F22_Composition2	$2.44\;\times \;10^{3}$	$2.50\;\times \;10^{3}$	$3.67\;\times \;10^{3}$	$5.99\;\times \;10^{3}$	$2.86\;\times \;10^{3}$	$3.14\;\times \;10^{3}$	$2.69\;\times \;10^{3}$	$4.07\;\times \;10^{3}$	$3.06\;\times \;10^{3}$	$2.76\;\times \;10^{3}$
F23_Composition3	$4.08\;\times \;10^{3}$	$4.11\;\times \;10^{3}$	$8.47\;\times \;10^{3}$	$4.66\;\times \;10^{4}$	$3.06\;\times \;10^{4}$	$1.54\;\times \;10^{4}$	$4.53\;\times \;10^{3}$	$4.69\;\times \;10^{4}$	$1.30\;\times \;10^{4}$	$6.36\;\times \;10^{3}$
F24_Composition4	$3.57\;\times \;10^{3}$	$3.65\;\times \;10^{3}$	$5.36\;\times \;10^{3}$	$5.20\;\times \;10^{4}$	$2.39\;\times \;10^{4}$	$1.65\;\times \;10^{4}$	$3.81\;\times \;10^{3}$	$4.42\;\times \;10^{4}$	$1.03\;\times \;10^{4}$	$3.05\;\times \;10^{3}$
F25_Composition5	$1.14\;\times \;10^{4}$	$2.85\;\times \;10^{4}$	$2.68\;\times \;10^{6}$	$1.38\;\times \;10^{10}$	$4.55\;\times \;10^{8}$	$6.61\;\times \;10^{8}$	$1.04\;\times \;10^{6}$	$8.38\;\times \;10^{9}$	$4.46\;\times \;10^{8}$	$1.35\;\times \;10^{4}$
F26_Composition6	$2.91\;\times \;10^{3}$	$2.91\;\times \;10^{3}$	$2.90\;\times \;10^{3}$	$2.94\;\times \;10^{3}$	$2.92\;\times \;10^{3}$	$2.90\;\times \;10^{3}$	$2.92\;\times \;10^{3}$	$2.92\;\times \;10^{3}$	$2.92\;\times \;10^{3}$	$2.90\;\times \;10^{3}$
F27_Composition7	$3.29\;\times \;10^{3}$	$3.29\;\times \;10^{3}$	$3.39\;\times \;10^{3}$	$4.10\;\times \;10^{3}$	$3.34\;\times \;10^{3}$	$3.62\;\times \;10^{3}$	$4.01\;\times \;10^{3}$	$3.57\;\times \;10^{3}$	$3.38\;\times \;10^{3}$	$3.43\;\times \;10^{3}$
F28_Composition8	$5.16\;\times \;10^{3}$	$1.35\;\times \;10^{4}$	$3.46\;\times \;10^{8}$	$9.75\;\times \;10^{9}$	$9.18\;\times \;10^{9}$	$9.32\;\times \;10^{8}$	$8.72\;\times \;10^{5}$	$9.62\;\times \;10^{9}$	$2.61\;\times \;10^{9}$	$5.73\;\times \;10^{4}$
F29_Composition9	$2.86\;\times \;10^{4}$	$2.92\;\times \;10^{4}$	$1.34\;\times \;10^{5}$	$5.04\;\times \;10^{6}$	$1.03\;\times \;10^{6}$	$1.32\;\times \;10^{6}$	$8.91\;\times \;10^{4}$	$5.40\;\times \;10^{6}$	$1.84\;\times \;10^{5}$	$2.90\;\times \;10^{4}$
F30_Composition10	$1.88\;\times \;10^{7}$	$1.89\;\times \;10^{7}$	$2.10\;\times \;10^{7}$	$5.34\;\times \;10^{9}$	$2.16\;\times \;10^{8}$	$1.84\;\times \;10^{8}$	$2.01\;\times \;10^{7}$	$2.73\;\times \;10^{9}$	$4.56\;\times \;10^{7}$	$1.90\;\times \;10^{7}$
**Friedman rank**	1.59	2.38	4.28	9.69	6.14	6.97	5.38	9.10	6.21	3.28

**Table 5:** CEC2022 (D=20) results: mean best fitness over 30 runs for all 10 algorithms. Best per row in bold. A lower Friedman rank is better.

Function	CCO-RL	CCO	GA	PSO	DE	GWO	HHO	RUN	EO	FGO
F1	$3.00\;\times \;10^{2}$	$3.00\;\times \;10^{2}$	$3.00\;\times \;10^{2}$	$3.01\;\times \;10^{2}$	$3.00\;\times \;10^{2}$	$3.01\;\times \;10^{2}$	$3.00\;\times \;10^{2}$	$3.01\;\times \;10^{2}$	$3.00\;\times \;10^{2}$	$3.00\;\times \;10^{2}$
F2	$4.12\;\times \;10^{2}$	$4.04\;\times \;10^{2}$	$4.53\;\times \;10^{2}$	$1.61\;\times \;10^{3}$	$4.38\;\times \;10^{2}$	$5.68\;\times \;10^{2}$	$5.26\;\times \;10^{2}$	$8.52\;\times \;10^{2}$	$4.58\;\times \;10^{2}$	$4.28\;\times \;10^{2}$
F3	$6.00\;\times \;10^{2}$	$6.00\;\times \;10^{2}$	$6.00\;\times \;10^{2}$	$6.00\;\times \;10^{2}$	$6.00\;\times \;10^{2}$	$6.00\;\times \;10^{2}$	$6.00\;\times \;10^{2}$	$6.00\;\times \;10^{2}$	$6.00\;\times \;10^{2}$	$6.00\;\times \;10^{2}$
F4	$8.44\;\times \;10^{2}$	$8.59\;\times \;10^{2}$	$9.35\;\times \;10^{2}$	$9.32\;\times \;10^{2}$	$9.25\;\times \;10^{2}$	$8.97\;\times \;10^{2}$	$9.11\;\times \;10^{2}$	$9.45\;\times \;10^{2}$	$9.07\;\times \;10^{2}$	$8.54\;\times \;10^{2}$
F5	$9.00\;\times \;10^{2}$	$9.00\;\times \;10^{2}$	$9.00\;\times \;10^{2}$	$9.00\;\times \;10^{2}$	$9.00\;\times \;10^{2}$	$9.00\;\times \;10^{2}$	$9.00\;\times \;10^{2}$	$9.00\;\times \;10^{2}$	$9.00\;\times \;10^{2}$	$9.00\;\times \;10^{2}$
F6	$5.24\;\times \;10^{4}$	$4.40\;\times \;10^{4}$	$9.07\;\times \;10^{4}$	$9.24\;\times \;10^{7}$	$1.58\;\times \;10^{7}$	$3.15\;\times \;10^{7}$	$4.52\;\times \;10^{5}$	$5.43\;\times \;10^{7}$	$1.13\;\times \;10^{5}$	$4.98\;\times \;10^{4}$
F7	$2.04\;\times \;10^{3}$	$2.04\;\times \;10^{3}$	$2.06\;\times \;10^{3}$	$2.43\;\times \;10^{3}$	$2.11\;\times \;10^{3}$	$2.10\;\times \;10^{3}$	$2.17\;\times \;10^{3}$	$2.24\;\times \;10^{3}$	$2.09\;\times \;10^{3}$	$2.07\;\times \;10^{3}$
F8	$2.23\;\times \;10^{3}$	$2.23\;\times \;10^{3}$	$2.23\;\times \;10^{3}$	$2.24\;\times \;10^{3}$	$2.24\;\times \;10^{3}$	$2.24\;\times \;10^{3}$	$2.23\;\times \;10^{3}$	$2.25\;\times \;10^{3}$	$2.23\;\times \;10^{3}$	$2.23\;\times \;10^{3}$
F9	$2.40\;\times \;10^{3}$	$2.40\;\times \;10^{3}$	$2.40\;\times \;10^{3}$	$2.63\;\times \;10^{3}$	$2.45\;\times \;10^{3}$	$2.50\;\times \;10^{3}$	$2.49\;\times \;10^{3}$	$2.58\;\times \;10^{3}$	$2.60\;\times \;10^{3}$	$2.41\;\times \;10^{3}$
F10	$2.71\;\times \;10^{3}$	$2.71\;\times \;10^{3}$	$2.76\;\times \;10^{3}$	$2.73\;\times \;10^{3}$	$2.71\;\times \;10^{3}$	$2.72\;\times \;10^{3}$	$2.83\;\times \;10^{3}$	$2.71\;\times \;10^{3}$	$2.71\;\times \;10^{3}$	$2.71\;\times \;10^{3}$
F11	$3.95\;\times \;10^{3}$	$3.80\;\times \;10^{3}$	$4.11\;\times \;10^{3}$	$5.70\;\times \;10^{3}$	$4.08\;\times \;10^{3}$	$4.23\;\times \;10^{3}$	$4.98\;\times \;10^{3}$	$1.20\;\times \;10^{4}$	$4.88\;\times \;10^{3}$	$3.71\;\times \;10^{3}$
F12	$3.12\;\times \;10^{3}$	$3.12\;\times \;10^{3}$	$3.14\;\times \;10^{3}$	$3.14\;\times \;10^{3}$	$3.12\;\times \;10^{3}$	$3.13\;\times \;10^{3}$	$3.17\;\times \;10^{3}$	$3.12\;\times \;10^{3}$	$3.13\;\times \;10^{3}$	$3.13\;\times \;10^{3}$
**Friedman rank**	2.12	1.71	5.58	8.83	5.58	6.67	7.00	8.25	5.92	3.33

**Table 6:** Friedman mean ranks (lower is better) of all 10 algorithms on each suite and overall (pooled 70 instances). Best per column in bold.

Algorithm	D=30 (CEC2017)	D=50 (CEC2017)	D=20 (CEC2022)	Overall
**CCO-RL**	1.62	1.59	2.12	1.69
CCO	1.76	2.38	1.71	2.01
FGO	4.10	3.28	3.33	3.63
GA	4.45	4.28	5.58	4.57
DE	5.48	6.14	5.58	5.77
HHO	6.10	5.38	7.00	5.96
EO	6.17	6.21	5.92	6.14
GWO	6.72	6.97	6.67	6.81
RUN	9.03	9.10	8.25	8.93
PSO	9.55	9.69	8.83	9.49

**Table 7:** Win/Tie/Loss counts of CCO-RL against each competitor (per-function mean comparison) with the Wilcoxon signed-rank *p*-value over all 70 instances.

CCO-RL vs.	D=30 (n = 29)	D=50 (n = 29)	CEC2022 (n = 12)	Total (n = 70)	Wilcoxon *p*
CCO	20/0/9	23/0/6	4/1/7	47/1/22	0.000
GA	27/0/2	27/0/2	12/0/0	66/0/4	<$10^{-4}$
PSO	29/0/0	29/0/0	12/0/0	70/0/0	<$10^{-4}$
DE	28/0/1	29/0/0	10/0/2	67/0/3	<$10^{-4}$
GWO	28/0/1	28/0/1	12/0/0	68/0/2	<$10^{-4}$
HHO	27/0/2	28/0/1	12/0/0	67/0/3	<$10^{-4}$
RUN	29/0/0	29/0/0	11/0/1	69/0/1	<$10^{-4}$
EO	29/0/0	29/0/0	11/0/1	69/0/1	<$10^{-4}$
FGO	26/0/3	22/0/7	10/0/2	58/0/12	<$10^{-4}$

**Table 8:** CEC2017 (D=100) results: mean best fitness over 30 runs, CCO-RL versus CCO. Best per row in bold. CCO-RL W/T/L =22/6/1 (Wilcoxon, α=0.05).

Function	CCO-RL	CCO
F1_BentCigar	$1.82\;\times \;10^{9}$	$1.13\;\times \;10^{9}$
F3_Zakharov	$1.47\;\times \;10^{5}$	$1.66\;\times \;10^{5}$
F4_Rosenbrock	$1.39\;\times \;10^{7}$	$3.15\;\times \;10^{7}$
F5_Rastrigin	$9.44\;\times \;10^{3}$	$1.12\;\times \;10^{4}$
F6_ExpandedScaffer	$6.44\;\times \;10^{2}$	$6.45\;\times \;10^{2}$
F7_LunacekBiRastrigin	$9.16\;\times \;10^{3}$	$9.49\;\times \;10^{3}$
F8_NonContRastrigin	$1.25\;\times \;10^{4}$	$1.26\;\times \;10^{4}$
F9_Levy	$1.18\;\times \;10^{4}$	$1.58\;\times \;10^{4}$
F10_Schwefel	$1.11\;\times \;10^{3}$	$1.14\;\times \;10^{3}$
F11_Hybrid1	$2.00\;\times \;10^{5}$	$2.75\;\times \;10^{5}$
F12_Hybrid2	$5.38\;\times \;10^{6}$	$2.39\;\times \;10^{7}$
F13_Hybrid3	$9.76\;\times \;10^{5}$	$7.15\;\times \;10^{6}$
F14_Hybrid4	$5.47\;\times \;10^{3}$	$1.27\;\times \;10^{4}$
F15_Hybrid5	$2.39\;\times \;10^{5}$	$1.01\;\times \;10^{6}$
F16_Hybrid6	$5.06\;\times \;10^{3}$	$1.68\;\times \;10^{4}$
F17_Hybrid7	$2.07\;\times \;10^{3}$	$2.07\;\times \;10^{3}$
F18_Hybrid8	$4.16\;\times \;10^{4}$	$4.96\;\times \;10^{5}$
F19_Hybrid9	$3.49\;\times \;10^{3}$	$7.12\;\times \;10^{3}$
F20_Hybrid10	$2.15\;\times \;10^{3}$	$2.19\;\times \;10^{3}$
F21_Composition1	$9.21\;\times \;10^{3}$	$1.21\;\times \;10^{4}$
F22_Composition2	$3.05\;\times \;10^{3}$	$3.17\;\times \;10^{3}$
F23_Composition3	$1.35\;\times \;10^{4}$	$1.57\;\times \;10^{4}$
F24_Composition4	$1.08\;\times \;10^{4}$	$1.31\;\times \;10^{4}$
F25_Composition5	$2.28\;\times \;10^{7}$	$4.53\;\times \;10^{7}$
F26_Composition6	$2.92\;\times \;10^{3}$	$2.92\;\times \;10^{3}$
F27_Composition7	$3.58\;\times \;10^{3}$	$3.66\;\times \;10^{3}$
F28_Composition8	$2.06\;\times \;10^{7}$	$2.30\;\times \;10^{7}$
F29_Composition9	$5.27\;\times \;10^{5}$	$2.91\;\times \;10^{5}$
F30_Composition10	$4.53\;\times \;10^{7}$	$4.49\;\times \;10^{7}$

**Table 9:** Mean wall-clock runtime per independent run (seconds), averaged over all functions of each suite, on the common hardware described in Section 5.2.

Algorithm	CEC2017 D=30	CEC2017 D=50	CEC2022 D=20
**CCO-RL**	6.61	7.56	3.71
CCO	5.36	6.41	3.52
GA	9.49	4.94	3.40
PSO	7.99	4.08	1.48
DE	9.91	4.78	3.78
GWO	10.20	4.50	3.56
HHO	21.91	7.95	5.33
RUN	7.69	5.25	2.48
EO	9.45	4.48	3.78
FGO	4.71	5.16	1.84

**Table 10:** Mean penalized objective (standard deviation in parentheses) over 30 runs on the three engineering design problems. Best mean per row in bold; CCO-RL is significantly better than CCO on all three (Wilcoxon, α=0.05).

Problem	CCO-RL	CCO
Pressure Vessel Design	5885.39(0.21)	5886.55(1.01)
Welded Beam Design	$1.861644\;(3.6\;\times \;10^{-8})$	$1.861676\;(4.7\;\times \;10^{-5})$
Tension/Compression Spring	$0.0126661\;(1.8\;\times \;10^{-6})$	$0.0126709\;(6.7\;\times \;10^{-6})$

## Data Availability

The source code, runner scripts and per-function result data that support the findings of this study are openly available in a public repository on Zenodo at https://doi.org/10.5281/zenodo.20610269. The repository contains the implementations of the CCO and CCO-RL, the experiment runners, the per-function result CSV files for all ten algorithms on every suite and the qualitative diagnostic figures.
